# Liver Transcriptome Changes in Zebrafish during Acclimation to Transport-Associated Stress

**DOI:** 10.1371/journal.pone.0065028

**Published:** 2013-06-10

**Authors:** Anusha K. S. Dhanasiri, Jorge M. O. Fernandes, Viswanath Kiron

**Affiliations:** Faculty of Biosciences and Aquaculture, University of Nordland, Bodø, Norway; University of California, Irvine, United States of America

## Abstract

Liver plays a key role during the stress acclimation, and liver transcriptome analysis of shipped zebrafish could reveal the molecular adjustments that occur in the organ. Transcriptional changes in liver were analyzed with a 44 K oligo array using total RNA from fish prior to transport and during a mock transport process - immediately after packing (0 h), at 48 and 72 h. Large numbers of genes related to a variety of biological processes and pathways were regulated, mainly during transport (at 48/72 h). Immediately after packing, transcripts of genes related to both gluconeogenesis and glycolysis were induced. During transport, induction of gluconeogenesis-linked genes and reduction of glycolysis-related genes may be supporting the increase in blood glucose levels. Inhibition of genes involved in fatty acid beta-oxidation may be pointing to the poor ability of fish to utilize energy from fatty acids, under transport conditions. Genes involved in some of the mechanisms that regulate body ammonia were also affected. Even though genes associated with certain transaminases were inhibited in liver, sustained glutamate deamination may have led to high ammonia accumulation in liver/body. Enhanced levels of gene transcripts in ubiquitination and MAPK signalling cascade and reduced levels of gene transcripts related to ROS generation via peroxisomal enzymes as well as xenobiotic metabolism may be signifying the importance of such cellular and tissue responses to maintain homeostasis. Furthermore, transcripts connected with stress and thyroid hormones were also regulated. Moreover, suppression of genes related to specific immune components may be denoting the deleterious impact of transport on fish health. Thus, this study has revealed the complex molecular -adjustments that occur in zebrafish when they are transported.

## Introduction

Live fishes that are transported worldwide undergo numerous stressful procedures. Firstly, pre-transport preparations such as collection, grading, netting, air exposure and packing are stressful to fish [1], [2]. Secondly, during transport, fish are confined at high packing densities in closed units, wherein the accumulating metabolic wastes can imperil fish [3], [4], [5]. The complexity of changes in water quality in these transport units is dependent on the interrelationship between parameters such as ammonia, nitrite, nitrate, oxygen, carbon dioxide and pH [6], [7], [8], [9]. Ammonia accumulating in water during transport can be one of the main inducers of stress [10], [11], [12], [13]. In addition, oxygen super saturation can trigger oxidative stress [14], [15], [16] even though this parameter is not known to elevate cortisol or ammonia levels [17]. Elevation of cortisol levels, a global response to stress, has been observed during transport [1], [2], [18], [19].

A variety of physiological and biochemical mechanisms in fish aid in their primary adaptation to stress, and these responses may become dysfunctional if stress is severe and long lasting [20], [21]. As pointed out in several studies, the liver plays a key role in homeostatic processes [22], [23], [24], thereby supporting adjustments during stressful conditions. This vital organ, where amino acid catabolism also takes place, provides the high energy needed by other tissues to regain homeostasis during stress [25], [26], [27]. Catabolism of amino acids in liver of fish is carried out primarily by transdeamination that generates ammonia [28]. On the other hand, the liver can also minimize the risk of ammonia intoxication by reducing ammonia production or/and converting ammonia to glutamine [29], [30]. Transcriptome analyses have revealed that a vast number of genes related to biological processes are regulated during stressful conditions [31], [32], [33]. These alterations are thought to be required to maintain homeostasis.

In the present study, the impact of transport stress was evaluated by profiling the liver transcriptome of zebrafish subjected to pre-transport procedures and to transport for up to 72 h. The aim was to understand the molecular adaptations linked to energy metabolism, response to ammonia accumulation, immune response, stress and thyroid hormone regulation and various other cellular and tissue responses that occur in zebrafish to maintain homeostasis.

## Methods

### Ethics Statement

The study was approved by the National Animal Research Authority (FDU) of Norway (Reference number: ID 2455). Rearing and handling of fish were carried out according to the guidelines of FDU (www.fdu.no; see “Forskrift om forsøk med dyr”).

### Fish Maintenance and Design of the Transport Experiment

Zebrafish (*Danio rerio*), of the size range 4–4.3 cm were maintained in a benchtop recirculation system (Aquatic Eco-Systems Inc., Apopka, Florida, USA) at the zebrafish rearing facilities of the University of Nordland, Bodø, Norway as described earlier [34]. Water quality parameters were monitored regularly and the average values recorded for total ammonia nitrogen (TAN), nitrate and pH were 0.042 mg l^−1^, 33.6 mg l^−1^ and 7.5, respectively. Fish were fed twice a day to apparent satiation on commercial flakes (TetraMin tropical flakes, Tetra Werke, Melle, Germany), and feeding was terminated 24 h prior to the mock transport.

The mock transport was performed in 5 l plastic bags filled with 1.5 l water, each bag carrying fish at a density of 30 per litre (45 per bag), as detailed in our previous publication [34]. The bags were charged with 3.5 l pure oxygen, following the ornamental fish industry practice [8]. A total of twelve bags were set up for the mock transport, which was conducted in a temperature-controlled dark room at 26°C for a duration of 72 h. Undisturbed fish from 4 individual tanks of the bench top system were sampled prior to the transport experiment, representing basal samples of unstressed fish. In addition, fish were collected from four randomly selected bags at 0 h (immediately after packing), 48 and 72 h after the start of transport. Henceforth, the term ‘during transport’ is used when referring to 48 and 72 h, and the term ‘during the transport process’ indicates changes at 0, 48 and 72 h. The bags that were opened for sampling at a particular time point were removed from the set up. Fish were terminally anaesthetized with a lethal dose (200 mg l^−1^) of tricaine methanesulfonate (MS222; Argent Chemical Laboratories, Redmond, USA), immediately frozen in liquid nitrogen and stored at −80°C for analysis of the whole body and liver ammonia (7 fish per sampling point), whole body cortisol (15 fish per sampling point) and for microarray analysis of liver transcriptome (20 fish per sampling point). For glucose measurements, blood was drawn using a capillary tube from 15 fish at the different time points.

Water samples were collected from all the bags that were part of the experiment, at each time point, and immediately stored at −40°C until analysing TAN, pH, nitrite and nitrate contents in it. Oxygen saturation and temperature in the bags that were opened at each time point were also recorded.

### Water Quality Analysis

TAN (sum of ionized and unionized ammonia) was determined, employing an ammonia high performance ion specific electrode (9512HPBNWP, Thermo Fisher Scientific, Beverly, USA), following the manufactureŕs instructions. Nitrite levels were analysed spectrophotometrically following the Griess method, whereas nitrate was measured with a nitrate combination ion specific electrode (3021, EDT Direct Ion Ltd., Dover, UK). Water pH and oxygen were measured using a pH meter (Mettler-Toledo International Inc, Columbus, USA) and portable dissolved oxygen meter (OxyGuard International A/S, Birkeroed, Denmark), respectively.

### Determination of Body Cortisol, Blood Glucose and Body/Liver Ammonia Levels

Whole body cortisol was analysed as described elsewhere [19] with the cortisol enzyme immunoassay kit (Cayman Chemical Company, Michigan, USA). Glucose levels in blood were measured using the FreeStyle Lite glucose monitoring system (Abbott Norge AS, Norway), following the produceŕs instructions.

Whole body and liver ammonia were determined with the ammonia assay kit (AA0100, Sigma-Aldrich Chemie GmbH, Munich, Germany), following the procedures described in our previous publication [34].

### RNA Extraction from Liver

Total RNA from 20 individual liver samples (5 fish from each of the 4 replicate tanks/bags at a time point) collected at each time point were extracted using the mirVana™ miRNA isolation kit (Ambion® Life Technologies, Carlsbad, USA). The female to male ratio of fish sampled at each time point was 3∶2. Equal amounts of RNA from 5 liver samples obtained from fish held in one tank/bag at a particular time point were pooled to obtain one biological replicate. Thus, 4 biological replicates were employed for the subsequent microarray analysis. By doing so, we were able to obtain representative samples from more fish at a particular time point. Even if microarray analyses performed on pooled samples may obscure to some extent the variation among individuals, inference of the results from most genes is not adversely affected by pooling [35] and pooling biological samples appropriately is statistically valid and efficient for microarray experiments [36]. The quality of RNA was assessed by agarose gel electrophoresis (individual liver RNA) and the Agilent 2100 Bioanalyzer (Agilent Technologies, Santa Clara, USA, pooled liver RNA). Its quantity was determined spectrophotometrically using the nanodrop ND-100 (NanoDrop Technologies, Inc., Wilmington, USA).

### Microarray Analysis on Liver

Single-color gene expression microarray analysis was performed using an Agilent Zebrafish Oligo Microarray (V3) (4×44 K) at the University Health Network Microarray Centre, Toronto, Canada (http://www.microarrays.ca/). For the analysis, 16 arrays that comprised of 4 biological replicates from 4 sampling points were employed. Microarray analysis was carried out following the protocol for Agilent’s one-color microarray-based gene expression analysis using cyanine 3 (Cy3) labeling. Microarray slides were scanned using the Agilent Microarray Scanner (model G2505B–C) and Feature Extraction Software 10.7.3. Raw data were first checked for overall quality using R (v2.14.1) with the Bioconductor framework and the Array Quality Metrics package. Then, they were imported into Genespring v12.1 (Agilent Technologies) for analysis. Standard normalizations and transformations, including median shift normalization to 80^th^ percentile and baseline transformations, were performed as recommended for Agilent single color microarrays. The data was then divided into 4 groups: basal, 0, 48 and 72 h and filtered to remove the probes that gave poor signals. Only probes that were in the upper 80^th^ percentile of the distribution of intensities in 100% of the samples (all the 4 biological replicates at a particular time point) in any of the 4 above mentioned groups (time points) were considered acceptable. Raw and processed data were submitted to Gene Expression Omnibus at NCBI (accession no: GSE44078). The filtered list was subjected to a second selection, and only probes with a fold change ≥2 in at least one of the possible 6 pairwise comparisons, between the time points, were retained. The resulting list was analysed using one way ANOVA and multiple testing correction was performed on the t-test P value with Benjamini-Hochberg FDR in Genespring v12.1. Post-hoc Tukey’s HSD (Honestly Significant Difference) test was employed to detect the differences at P<0.05 between the groups of interest.

Significantly regulated probes at 0, 48 and 72 h, compared to basal levels, were subjected to GO enrichment analysis using the GOEAST web based software (http://omicslab.genetics.ac.cn/GOEAST/index.php) [37] with the Agilent Zebrafish V3 gene expression microarray as reference. GOEAST uses - hypergeometric method for statistical analysis and the P values were corrected with Benjamini –Yekutieli False Discovery Rate (FDR). Some gene annotations that were not provided by GOEAST were obtained from DAVID (http://david.abcc.ncifcrf.gov/) [38]. Pathway enrichment analysis was carried out using GeneSpring v12.1 after importing curated pathways for zebrafish from the WikiPathways portal (http://www.wikipathways.org) and using the Agilent Zebrafish V3 gene expression microarray as reference. Heat maps were prepared using the MultiExperiment Viewer-MeV online tool (http://www.tm4.org/mev/) [39]. For the preparation of heat maps of expression profiles of genes in representative GO terms, genes which showed significant differences between at least one of the time points (0, 48 and 72 h) and basal levels were used.

### Validating Microarray Data using Quantitative Real Time PCR (qPCR)

The expression of 6 genes (stearoyl-CoA desaturase (delta-9-desaturase), scd; glutamic-oxaloacetic transaminase 2 (mitochondrial)/aspartate aminotransferase 2, got2a; ubiquitin-conjugating enzyme E2H (UBC8 homolog, yeast), ube2h; glutathione peroxidase 4b, gpx4b; MAP kinase-interacting serine/threonine kinase 2b, mknk2b and 11β-hydroxysteroid dehydrogenase 2, hsd11b2), from among the list of up- or down-regulated transcripts were profiled using qPCR. Primers were designed to span at least one intron/exon border, using the primer designing tool Primer-BLAST at NCBI (http://www.ncbi.nlm.nih.gov/). Quality of the primers was checked using PREMIER Biosoft online tool (http://www.premierbiosoft.com/). The primers used in this study are listed in Table S1 in File S1. RNA from liver samples of 16 individual fish (selected out of the 20 utilized for microarray analysis) were used for qPCR analysis. cDNA was synthesized from 1 µg of RNA using QuantiTect reverse transcription kit (Qiagen AB, Sollentuna, Sweden), following the manufacturer’s protocol. qPCR was performed with 1∶15 dilution of the above cDNA, with Fast SYBR® Green PCR master mix (Applied Biosystems, Warrington, UK) using the StepOne Plus™ instrument (Applied Biosystems). The thermal cycling profile was as follows: initial activation of 20 s at 95°C, followed by 40 cycles of 3 s at 95°C and 30 s at 60°C. Six-point standard curves were prepared from the cDNA obtained from five-fold serially diluted pooled RNA of all the samples. These standard curves were used to estimate the PCR efficiency of each primer set for each gene as described by Fernandes et al. [40], [41] and the efficiencies of all the genes were above 98% (Table S1 in File S1). Negative reverse transcriptase control and no template control were included. All reactions were carried out in duplicate and Cq values were averaged. The geNorm [42] normalization factors corresponding to the geometric mean of the relative quantities of two reference genes, (e*longation factor 1-alpha,* eef1a and *beta-2-microglobulin*, b2m) were used for relative quantification, as reported in Fernandes et al. [43].

### Statistical Analysis

The data on the whole body cortisol, blood glucose, whole body and liver ammonia were analyzed using one way ANOVA followed by Tukey’s post-hoc test. All pertinent assumptions (normality of distribution and equality of variance) were checked before performing each analysis and transformations of data (cortisol, whole body and liver ammonia) were carried out whenever necessary. Correlation analysis was performed to understand the relationship between microarray and qPCR data. Graph Pad Prism v5.0 software (Graphpad Software Inc., La Jolla, USA) was used for the statistical analysis and the significant differences are reported when P<0.05.

## Results

### Indicators of Water Quality

TAN surged from 0.10 (0 h) to 18.13 (48 h) and 32.77 (72 h) mg l^−1^ (Table S2 in File S1). Water nitrate increased from 4. 31 (0 h) to 6.77 (48 h) and 9. 69 (72 h) mg l^−1^ and nitrite was elevated from 0.008 (0 h) to 0.04 (48 h) and 0.039 (72 h) mg l^−1^ (Table S2 in File S1). On the other hand, water pH decreased from 7.67 (0 h) to 6.82 (48 h) and 6.55 (72 h) (Table S2 in File S1).

### Physiological Indicators Measured during the Transport Process

Cortisol level was significantly higher in the transported fish compared to that in the fish prior to transport (basal; Table 1). At 48 h of transport, whole body cortisol levels were significantly lower compared to the values immediately after packing (0 h). Blood glucose levels were significantly higher only at 72 h of transport compared to the values prior to transport (Table 1). Whole body and liver ammonia levels (Table 1) were significantly higher at 48 and 72 h of transport compared to the values prior to transport (basal) and immediately after packing (0 h).

**Table 1:** Physiological parameters measured in zebrafish at various stages during the transport process and prior to transport.

Physiological parameter	Time point			
	Basal	0 h	48 h	72 h
Whole body cortisol (ng g^−1^)	2.51±0.51^a^	29.83±3.76^b^	14.36±2.3^c^	22.37±3.49^bc^
Blood glucose (mmol l^−1^)	1.52±0.15^a^	1.96±0.15	1.87±0.16	2. 42±0.23^b^
Whole body ammonia (µg g^−1^)	32.17±4.08^a^	50.21±8.25^a^	100.3±13.56^b^	153.7±15.92^b^
Liver ammonia (µg g^−1^)	38.35±5.08^a^	42.48±5.03^a^	140.4±25.85^b^	182.5±11.77^b^

Whole body cortisol (n = 15), blood glucose (n = 15), whole body ammonia (n = 7) and liver ammonia (n = 7) were analyzed prior to transport (basal), immediately after packing (0 h), and at 48 and 72 h of transport.

Different letters indicate statistically significant differences among the different time points (P<0.05).

Values are given as means ± s.e.m.

### Liver Transcriptome Profiling

**Figure 1:**
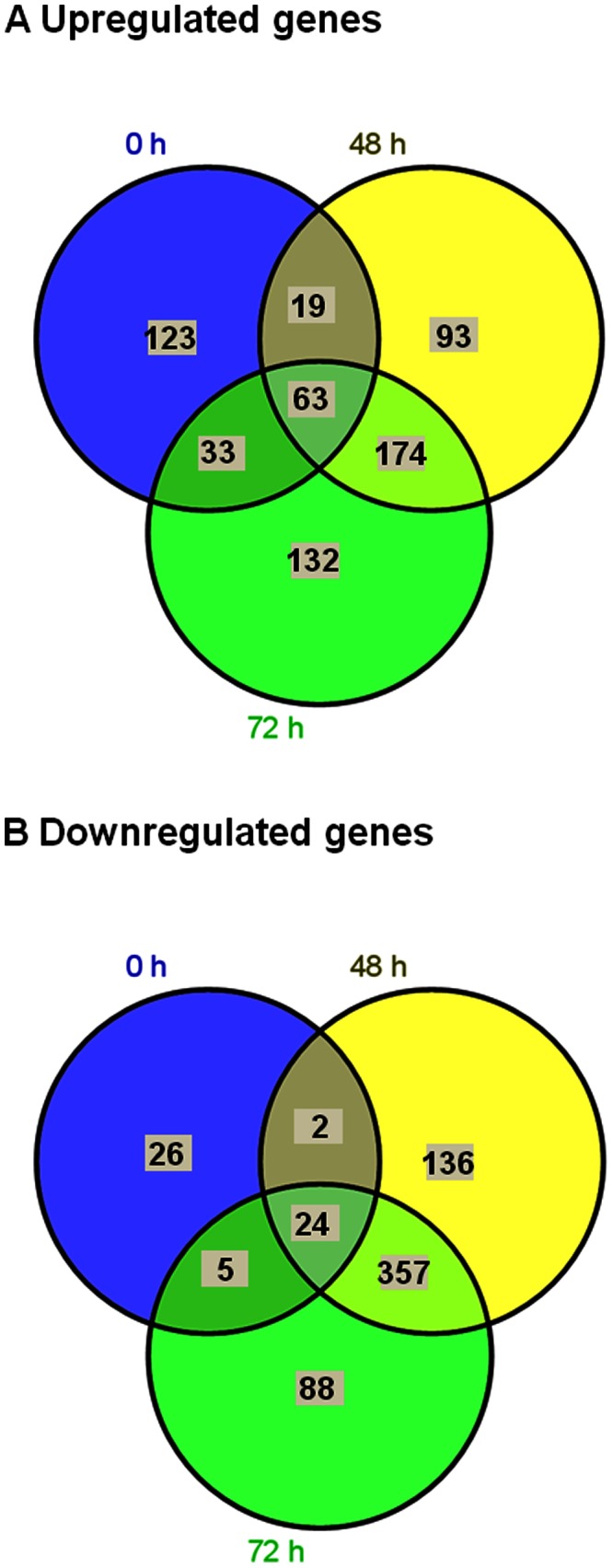
Venn diagrams indicating the differentially expressed genes in the zebrafish liver during the transport process. The number of upregulated (A) and downregulated (B) genes immediately after packing (0 h) and at 48 and 72 h during transport compared to the values prior to transport (basal) are marked inside each circle. The genes which have ≥2 fold change in at least one of the pair wise comparisons (between the time points) and significantly altered (P<0.05) compared to basal values are included for each time point.

During the transport process, expression of 637 genes (Fig. 1, Table S3 in File S1) were upregulated and that of 638 genes (Fig. 1, Table S4 in File S1) was downregulated compared to the expression of the corresponding genes prior to transport. Most of the gene regulation occurred during transport rather than immediately after pre-transport procedures.

### Gene Ontology (GO) Enrichment Analysis

**Figure 2:**
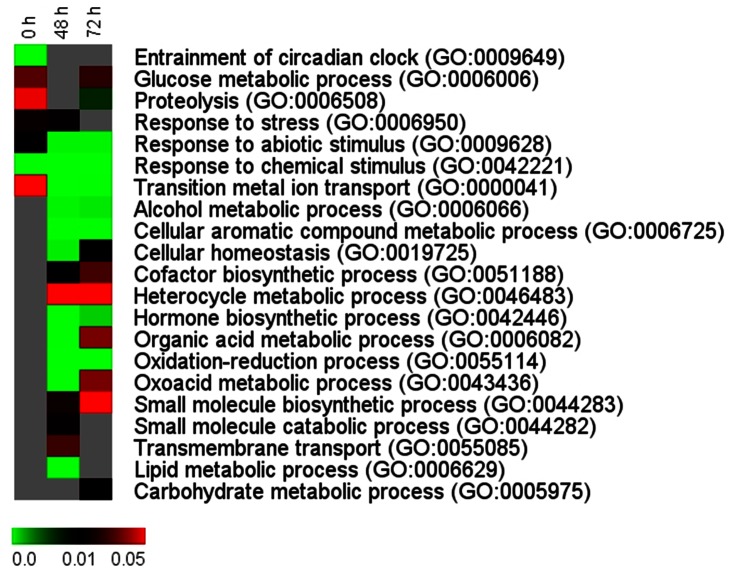
Heat maps from GO enrichment analysis of differentially regulated genes during the transport process. Significantly regulated genes - at each of the time point [immediately after packing (0 h), and at 48 and 72 h during transport] compared to the values prior to transport (basal) - were subjected to GO enrichment analysis. Representative biological process GO terms with P<0.05 were selected mostly from 3^rd^ or 4^th^ levels of GO trees in at least one of the time points. Columns and rows in the heat map indicate time points and GO terms, respectively. Color scale represents P values of enrichment test and gray cells indicate P>0.05.

To interpret the transcriptional changes that occur in liver during the transport process, differentially regulated genes at 0, 48 and 72 h, compared to the expression of the respective genes prior to transport (≥2 fold change and P<0.05) were subjected to GO enrichment analysis. GO terms representing the biological processes, selected mostly from 3^rd^ or 4^th^ GO levels, are displayed in Fig. 2.

GO enrichment analysis indicated that while only 33% (7/21) of the GO categories were significantly enriched immediately after packing (0 h), most of the GO terms (20/21) were overrepresented during transport (at 48 and/or 72 h; Fig. 2). The GO terms that were significantly enriched both after packing as well as during transport included, among others, glucose metabolic process, proteolysis, response to stress, abiotic stimulus and chemical stimulus. Several metabolic processes - those of lipid, carbohydrate and oxidation-reduction and cellular homeostasis - were also among the GO terms that were enriched during transport.

### Pathway Enrichment Analysis

**Figure 3:**
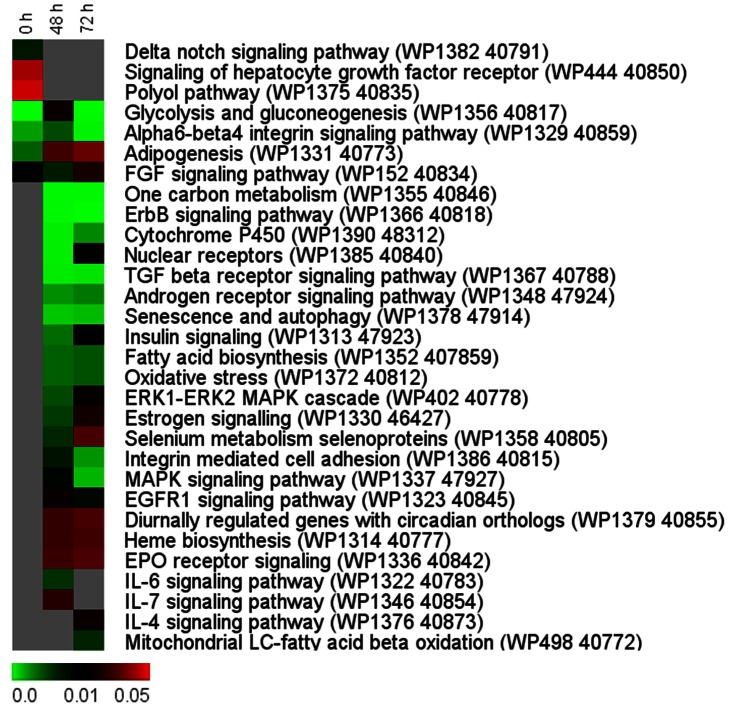
Heat maps from pathway enrichment analysis of differentially regulated genes during the transport process. Significantly regulated genes - at each of the time point [immediately after packing (0 h), and at 48 and 72 h during transport] compared to the values prior to transport (basal) - were subjected to pathway enrichment analysis. Pathways which showed an enrichment of P<0.05 in at least one of the time points are displayed. Columns and rows in the heat map indicate time points and pathways, respectively. Color scale represents P values of enrichment test and gray cells indicate P>0.05.

Pathway enrichment analysis on differentially regulated genes at 0, 48 and 72 h compared to their expression prior to transport indicated that most of the pathways 27/30 (90%) are significantly enriched at 48 and/or 72 h (Fig. 3); only a few pathways (23%) were significantly enriched at 0 h. Pathways of glycolysis and gluconeogenesis were enriched at all the time points during the transport process, while pathway of fatty acid biosynthesis were enriched only during transport. Some other pathways, including those of senescence and autophagy, oxidative stress, cytochrome P450, ERK1-ERK2 MAPK cascade and MAPK signaling were enriched during transport (Fig. 3). Several genes related to ERK1-ERK2 MAPK cascade including *v-fos FBJ murine osteosarcoma viral oncogene homolog*, *fos*; *jun B proto-oncogene like*, *junbl*; *ribosomal protein S6 kinase a, polypeptide 1*, *rps6ka1*; and *epidermal growth factor receptor a*, *egfra*; and MAPK signaling including *death-associated protein 6*, *daxx* were upregulated during the transport process (Table S3 in File S1).

### Representative Genes Regulated during the Transport Process - Related to Enriched GO Categories

**Figure 4:**
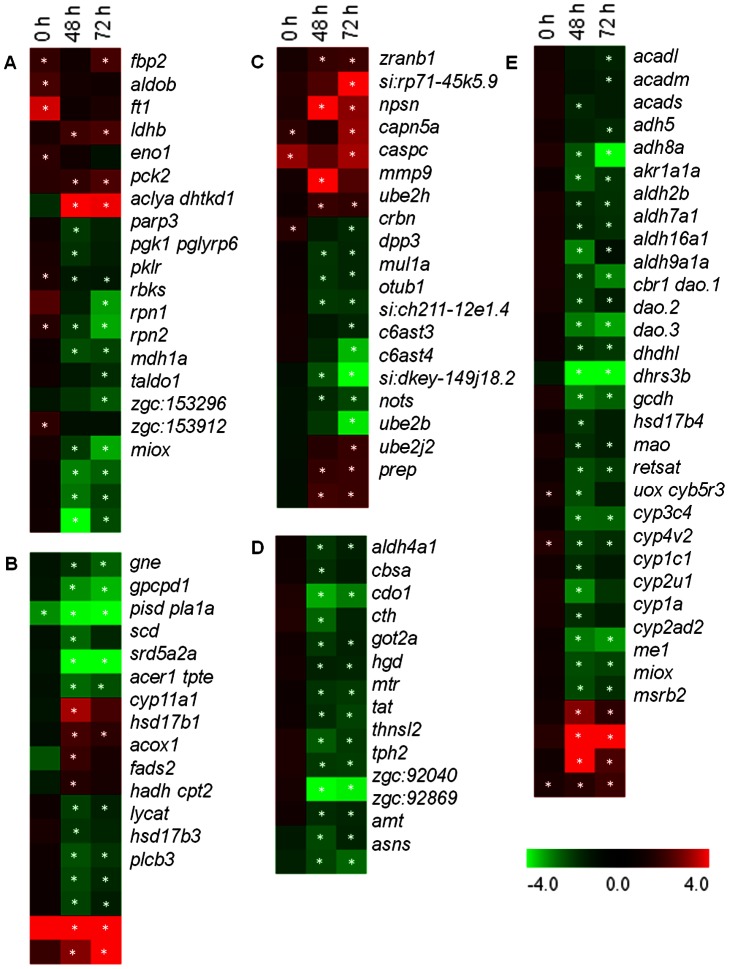
Expression profiles of representative genes in selected GO categories. (A) Carbohydrate metabolic process; (B) Lipid metabolic process; (C) Proteolysis; (D) Amino acid metabolic process; and (E) Oxidation-reduction process. Significantly regulated genes –in at least one of the time points [immediately after packing (0 h), and at 48 and 72 h during transport] compared to the values prior to transport (basal) - are displayed in the heat maps. Columns and rows in the heat map indicate time points and genes, respectively. Color scale represents fold changes of gene expression. Genes belonging to more than one GO category is shown only once under a particular GO term. *indicates significant differences (P<0.05) at a particular time point compared to the expression of the respective genes prior to transport (basal).

**Figure 5:**
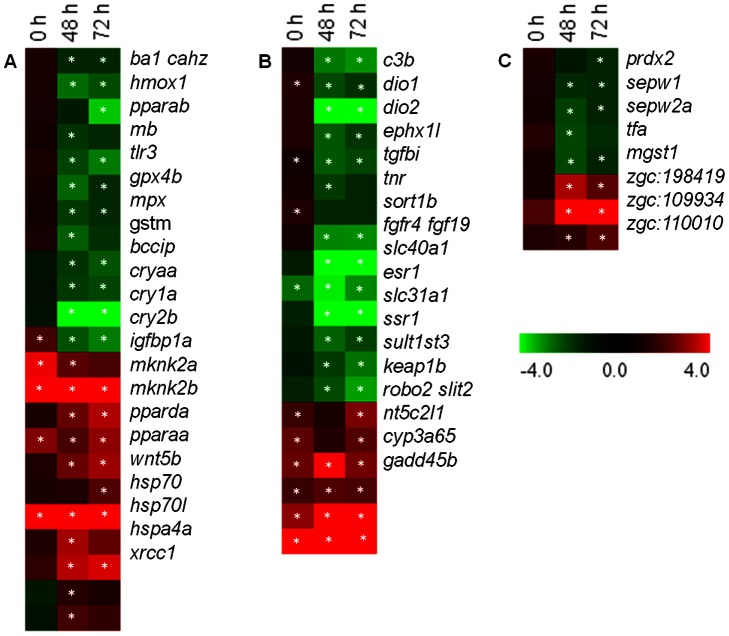
Expression profiles of representative genes in selected GO categories. (A) Response to stress; (B) Response to chemical stimulus; and (C) Cellular homeostasis. Significantly regulated genes – in at least one of the time points [immediately after packing (0 h), and at 48 and 72 h during transport] compared to the values prior to transport (basal) - are displayed in the heat maps. Columns and rows in the heat map indicate time points and genes, respectively. Color scale represents fold changes of gene expression. Genes belonging to more than one GO category is shown only once under a particular GO term. *indicates significant differences (P<0.05) at a particular time point compared to the expression of the respective genes before transport (basal).

The gene subsets from among the extensive list of differentially expressed genes that were impacted during the transport process are shown in heat maps. Genes that are associated with GO terms are categorized under metabolic processes (Fig. 4), response to stimuli and homeostasis (Fig. 5). Only the genes presented under a particular GO term in the respective heat maps are considered henceforth.

### Genes Involved in Metabolic Processes

#### Carbohydrate metabolic process

At 0 h, 35% of the genes related to carbohydrate metabolism were significantly regulated (mostly upregulated), while during transport, the percentage was higher (80%, mostly downregulated; Fig. 4A). Immediately after packing (0 h) genes linked to glycolysis/gluconeogenesis pathways- *aldolase b*, *aldob*; *fructose-1,6-bisphosphatase 2*, *fbp2*; *enolase 1, (alpha)*, *eno1* and *malate dehydrogenase 1a, NAD*, *mdh1a* - were significantly upregulated compared to the expression of the respective genes prior to transport (basal). All the other genes under this category were not altered during transport, except *fbp2* that was upregulated even at 72 h. Genes related to glycolysis such as *pyruvate kinase*, *pklr* and *phosphoglycerate kinase 1*, *pgk1* that were significantly upregulated after packing were downregulated during transport. *Lactate dehydrogenase B4*, *ldhb* and *phosphoenolpyruvate carboxykinase 2*, *pck2* were upregulated during transport.

#### Lipid metabolic process

Genes related to lipid metabolism, except *phosphatidylserine decarboxylase*, *pisd* were unaltered at 0 h (Fig. 4B). However, during transport they were significantly affected, mostly downregulated. Genes related to lipid biosynthesis including *stearoyl-CoA desaturase (delta-9-desaturase)*, *scd*; *glucosamine (UDP-N-acetyl)-2-epimerase/N-acetylmannosamine kinase*, *gne*; *fatty acid desaturase*, *fads2*; and *pisd*; and fatty acid metabolic process including *hydroxyacyl-Coenzyme A dehydrogenase*, *hadh*; and *acyl-Coenzyme A oxidase 1, palmitoyl*, *acox1* were significantly downregulated.

#### Proteolysis

While only few of the genes related to proteolysis were significantly regulated at 0 h, all of them were significantly controlled during transport (Fig. 4C). Genes related to ubiquitin-proteasome pathway including *zinc finger, RAN-binding domain containing 1*, *zranb1*; *ubiquitin-conjugating enzyme E2H (UBC8 homolog, yeast)*, *ube2h*; *ubiquitin-conjugating enzyme E2B (RAD6 homolog)* and *E2, J2 (UBC6 homolog, yeast)*, *ube2b* and *ube2j2*, respectively were significantly upregulated at 48 and/or 72 h of transport.

#### Cellular amino acid metabolic process

During transport, genes related to amino acid metabolism were significantly downregulated, whereas immediately after packing no significant changes were observed (Fig. 4D). The genes for enzymes (transaminases) that participate in the formation of glutamate from L-aspartate, L-tryptophan and/or L-phenylalanine and L-tyrosine such as *glutamic-oxaloacetic transaminase 2 (mitochondrial)/aspartate aminotransferase 2*, *got2a* and *tyrosine transaminase*, *tat* were downregulated during transport.

#### Oxidation reduction process

Genes related to oxidation reduction process were significantly regulated during transport, while only three of them were affected at 0 h (Fig. 4E). Genes in several oxidation-reduction reactions include those related to fatty acid metabolism, coenzyme binding, FAD (flavin adenine dinucleotide) binding and enzyme activities using NAD (nicotinamide adenine dinucleotide) or NADP (nicotinamide adenine dinucleotide phosphate). A number of genes in cytochrome P450 family, including, *cytochrome P450, family 1 subfamily A*, *cyp1a*; genes related to D-amino-acid oxidase activity such as *D-amino-acid oxidase 1, 2* and *3*, *dao.1*, *dao.2* and *dao.3*, respectively; and *urate oxidase*, *uox* were downregulated during transport. Moreover, genes in beta oxidation of fatty acids- *acyl-Coenzyme A dehydrogenase, long chain*, *acadl*; *acyl-Coenzyme A dehydrogenase, C-4 to C-12 straight chain*, *acadm*; and *acyl-Coenzyme A dehydrogenase, C-2 to C-3 short chain*, *acads* were also downregulated at 48/and 72 h of transport.

### Genes Involved in Response to Stress, Chemical Stimuli and Homeostatic Process

#### Response to stress

During transport, all the genes related to the GO term, response to stress, were significantly regulated, while immediately after packing only a few were upregulated (Fig. 5A). Genes related to antioxidant activity including *myeloid-specific peroxidase*, *mpx*; *glutathione peroxidase 4b*, *gpx4b*; and *glutathione S-transferase M*, *gstm*; heme degradation such as *heme oxygenase (decycling) 1*, *hmox1*; and response to bacterium including *toll-like receptor 3*, *tlr3* were significantly downregulated at 48 and/or 72 h of transport. Although during transport both *MAP kinase-interacting serine/threonine kinase 2a* and *2b*, *mknk2a* and *mknk2b* were upregulated, at 0 h only *mknk2b* was upregulated.

#### Response to chemical stimulus

Genes related to the GO term, response to chemical stimulus, were significantly regulated during transport, except a lysosomal membrane protein associated gene *sortilin 1b, sort1b*. However, at 0 h, only 50% of this set, including *sort1b*, were significantly regulated (Fig. 5B). Most of the genes including *deiodinase, iodothyronine, type 1* and *II*, *dio1* and *dio2* and *complement component C3B*, *c3b* were significantly downregulated during transport.

#### Cellular homeostasis

All the genes related to cellular homeostasis were significantly regulated during transport, while none of them were regulated at 0 h (Fig. 5C). Genes related to cell redox homeostasis, such as *peroxiredoxin 2*, *prdx2* and *microsomal glutathione S-transferase 1*, *mgst1* were significantly downregulated at 48 and/or 72 h transport.

### Other Immune and Stress-related Genes Regulated during the Transport Process

In addition to the aforementioned genes related to the enriched GO categories, several other immune and stress related genes were significantly regulated during the transport process (Table S3 and S4 in File S1). Immune genes such as suppressor of *cytokine signaling 8*, *socs8*; and *secreted immunoglobulin domain*, *sid4* were upregulated at 0 h and *interferon regulatory factor 11*, *irf11* was downregulated during transport. Further, *11β-hydroxysteroid dehydrogenase 2*, *hsd11b2* that is essential for cortisol regulation was upregulated at 0 h.

### Validation of Microarray Results using qPCR

**Figure 6:**
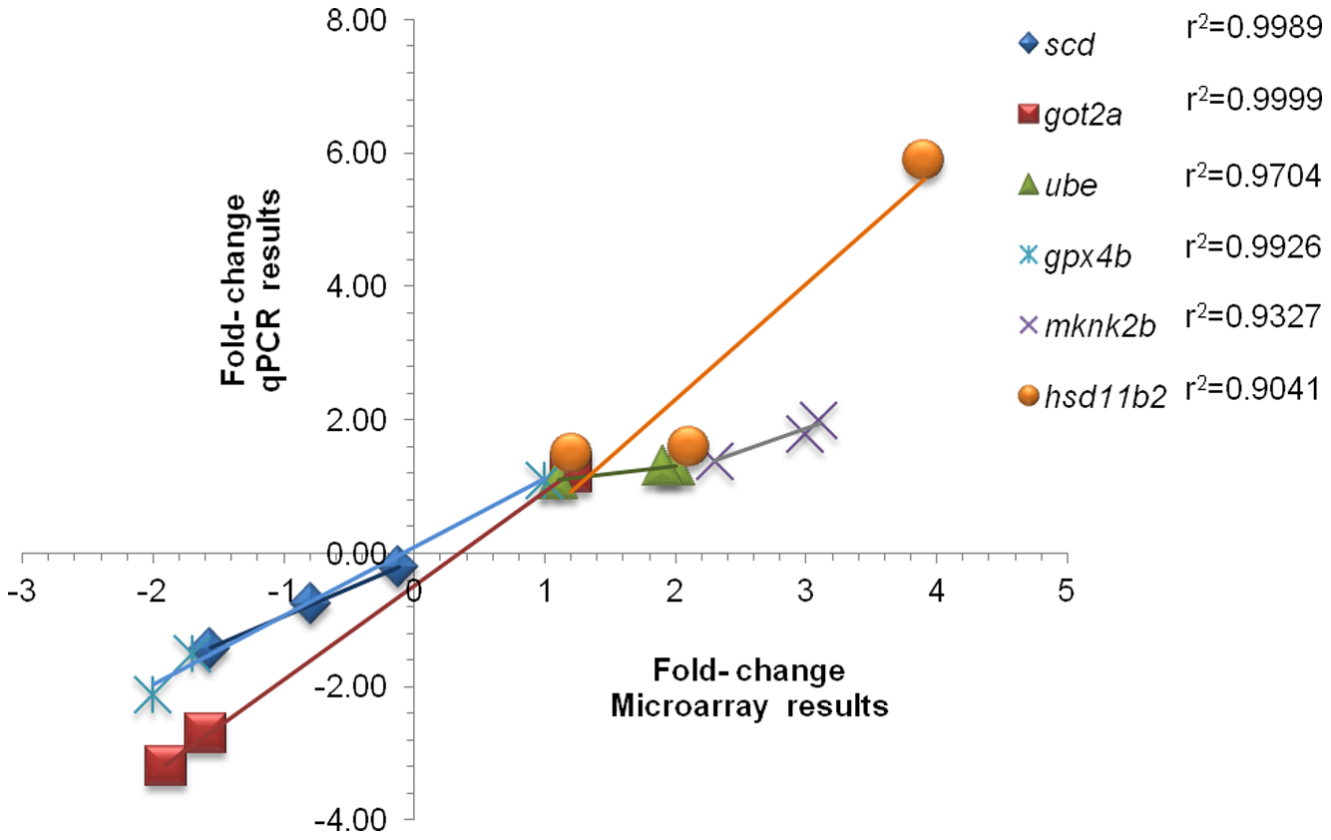
Validation of microarray results by quantitative real time PCR. Correlation plots indicating the relationship between qPCR results (fold change; Y- axis)) of six selected genes and the corresponding data from microarray analysis (X- axis). Fold changes of genes immediately after packing (0 h), and at 48 and 72 h during transport compared to the values prior to transport (basal) are displayed in the figure. Note that the fold changes for *scd* are 1/10^th^ of the actual changes.

The six target genes, *scd*, *got2a*, *ube2h*, *gpx4b, mknk2b* and *hsd11b2* selected for validation of microarray results, were representative of those which were either up- or down-regulated during the transport process compared to those prior to transport (basal). A high statistical correlation (r^2^>0.90) between qPCR and microarray results confirmed the validity of the microarray data (Fig. 6).

## Discussion

Pre-transport procedures as well as transport itself are stressful to fish that are being shipped [1], [8], [18], [19]. In the present study, procedures such as handling, netting and exposure to air that are unavoidable during packing have induced a stress response in zebrafish, as indicated by the peak in cortisol levels. During transport too, the cortisol levels were significantly higher compared to the basal value. However, the glucose levels increased significantly only at the 72 h time point. This extended (cortisol) or delayed (glucose) stress response during transport could be attributed mainly to the interaction between various physical and chemical stressors including pre-transport procedures, confinement to closed systems with high packing densities, oxygen super saturation and water quality changes (mainly ammonia accumulation). Here we presume that short-term starvation used in the present study (1 day prior to transport and 3 days during transport) may not have influenced the cortisol levels as similar (48 h starvation in rainbow trout, *Oncorhynchus mykiss* [44]) or long-term starvation (14 days in channel catfish, *Ictalurus punctat*us [45]) has not elevated this primary stress response. There were marked changes in the liver transcriptome as a result of the transport process, mainly at 48 and 72 h, pointing to the complexity of mechanisms underlying the stress response.

### Adjustments in Energy Metabolism

Metabolic alterations are key adaptive responses [25] to stress, and regulation of energy metabolism in liver during stress response has been observed in several fishes [27], [46], [47], [48]. Enhanced production of glucose in the liver is vital during stressful conditions, to compensate for the high energy demand needed to regain homeostasis. In the present study, glucose levels were elevated only at the 72 h time point. On the other hand, in jundia, *Rhamdia quelen* [49] and matrinxa, *Brycon cephalus* [50] blood glucose levels that were elevated as early as 4 h during transport returned to pre-stress levels by 24 and 96 h, respectively, after the transport. Rapid generation of glucose occurs through glycogenolysis, whereas long-term generation is through gluconeogenesis [25], [26]. Genes related to both gluconeogenesis and glycolysis (e.g. *eno1*, *pgk1*, *aldob* and *mdh1a*) as well as genes that code for rate limiting enzymes in gluconeogenesis (*fbp2*) and glycolysis (*pklr*) were upregulated by the pre-transport procedures during packing, but this was not reflected in the measured blood glucose levels. In rainbow trout liver too, increase in transcripts related to gluconeogenesis/glycolysis were not associated with changes in glucose levels, at 1 h following handling stress [33]. During transport, *pklr* and *pgk1* were inhibited and *eno1*, *aldob* and *mdh1a* were unaltered, possibly indicating a reduction in liver glycolysis. On the other hand, genes that code for key regulatory enzymes in gluconeogenesis (*pck2* at 48 and 72 h; *fbp2* at 72 h) and gene that codes for lactate dehydrogenase, which catalyzes the pyruvate synthesis from lactate (*Idhb*) were upregulated, likely pointing to an increase in gluconeogenesis using lactate as a substrate, mainly at 72 h. The genes linked to gluconeogenesis and glycolysis may be associated with the high blood glucose levels at 72 h.

Lipids are a valuable source of energy for fish and stress induced alterations of lipid metabolism are reported in several fishes [47], [48]. In the present study, several genes involved in beta oxidation of fatty acids including *acadl*, *acadm*, *acads*, *hadh* and *acox1* were inhibited in liver of zebrafish that were transported, possibly indicating the reduced energy supply from fatty acids. Utilization of fatty acids during stress is different among fish species [25]. Contradictory to our observations, during the early (6 h) hours of confinement, stress response in gilthead sea bream (*Sparus aurata*) liver was observed as an increase in transcripts linked to lipid metabolism [31]. During the present study, fish under transport stress were characterized by an inhibition of genes related to biosynthetic process of fatty acids (*scd* and *fads2*), phospholipids (*pisd*) and lipopolysaccharide (*gne*). Comparable to our results, *fads2* transcripts were downregulated in larval zebrafish subjected to temperature stress [51]. Genes linked to lipid metabolism were downregulated in both zebrafish [52] and rainbow trout [53] starved for 21 days and in Atlantic salmon, *Salmo salar* that were unfed for 28 days [54]. However, 4-day starvation period employed during the present study is very much shorter than in the above-mentioned studies. Therefore, further investigations are needed to ascertain the effect of this essential practice on transported fish. It needs to be emphasized that starvation is adopted to keep the nitrogen loading minimum during transport. The transcriptional changes related to lipid metabolism observed in the current study may be pointing to an impaired ability of transported fish to utilize energy from fatty acids.

### Response to Ammonia Accumulation

As ammonia content in fish body increases, its production decreases, excretion gets regulated and/or the conversion to a less toxic product glutamine occurs [30], [34], [55], [56]. Catabolism of amino acids in liver of fish is primarily carried out via transdeamination, during which an amino group of variety of amino acids is transferred to α-ketoglutarate by transaminases to form glutamate, which is then deaminated by glutamate dehydrogenase (GDH) to yield ammonia (NH_4_^+^) [28]. In the present study genes encoding for transaminases such as *got2a* and *tat* were downregulated during transport, possibly affecting the enzymes involved in production of glutamate. A reduction in glutamate would be necessary to reduce ammonia production. However, we have observed high ammonia levels in liver and whole body (Table 1) of zebrafish at the same time points in the present as well as in our previous studies [34]. This implies that a sustained glutamate deamination - occurring as a result of the delinking of the two arms of transdeamination (transamination and deamination) [57] - could have continued the ammonia production in the liver of zebrafish during transport. Further, as the water ammonia levels were high (Table S2 in File S1), the ammonia excretion rates would have reduced, leading to a surge in the body and liver ammonia. High ammonia levels in the body could have increased the glutamine synthetase activity (including that in the liver) to detoxify ammonia to glutamine [34]. Thus, based on this study and the previous report [34], it could be stated that during transport, the liver of fish carries out its function in detoxifying ammonia to glutamine.

### Cellular and Tissue Responses

Stress responses of cells and tissues, including their acclimation, repair or elimination of damaged cells or molecules, will ultimately determine the survival of an organism [58].

Among the pathways that control these responses, ubiquitin-proteasome pathway is crucial to selectively degrade proteins so as to regulate most cellular processes (including transcription, cell cycle progression, differentiation, development, immune responses), and to control the cellular response to stress and extracellular effectors [59]. The current study identified several genes related to ubiquitination including those in ubiquitin-protein ligase activity, *zranb1*; and genes that code for ubiquitin-conjugating enzymes, E2H (*ube2h*), E2B (*ube2b*) and E2, J2 (*ube2j2*) that were upregulated at 48 and/or 72 h of transport, pointing to a possible increase in ubiquitination during transport. ER-associated protein degradation (ERAD) by cytosolic ubiquitin-proteasome system is an essential mechanism that prevent potentially lethal aggregation of protein (misfolded or damaged proteins) in the endoplasmic reticulum [60]. Therefore, enhanced ubiquitination would be pertinent to augment ERAD and also to control numerous cellular processes [60], [61]. Further, an increase in transcripts linked to ERAD has been observed in liver during confinement stress in gilthead sea bream [31]. Thus, the upregulation of the ubiquitination-associated genes observed in the present study may be pointing to their involvement in regaining homeostasis during transport.

Transport stress arising from oxygen super saturation and ammonia in water can cause oxidative stress in fish [14], [15], [62], [63]. However, in the present study, genes involved in ROS generation through peroxisomal enzymes [64] including acyl-CoA oxidases (*acox1*); amino acid oxidase (*dao.1, dao.2* and *dao.3*); and urate oxidase (*uox*) were downregulated during transport, when both ammonia levels as well as oxygen super saturation in water were high (Table S2 in File S1). On the other hand, genes related to antioxidant activity (*mpx*, *gpx4b*, *gstm*, *prdx2*, and *mgst1*) were inhibited at 48 and/or 72 h transport. Further, the gene that codes for Hmox1, a protein involved in heme degradation, that is known to have a protective role against oxidative stress in mammals [65] were also downregulated at 72 h. These observations suggest lower ROS scavenging activity, perhaps associated with the reduction of ROS generation, that is indicated by the inhibition of the transcripts of peroxisomal enzymes. Furthermore, several genes in cytochrome P450 family including *cyp1a*, which are known to play a vital role in xenobiotic and endobiotic metabolism [66] were downregulated. In agreement with the results of the present work, another study on liver of gilthead sea bream during the confinement stress also indicated inhibition of transcripts in cytochrome P450 [31]. Activation of cytochrome P450 enzymes can lead to production of ROS [67]. Therefore, reduced transcripts possibly help in keeping the ROS generation low during transport. Thus, the molecular responses observed in the liver, which also partakes in detoxification, may be suggestive of the prevalence of lower oxidative stress or a response directed towards achieving ROS balance.

Eukaryotic cells regulate gene expression via MAPK (mitogen-activated protein kinase) signalling cascade under various stressful conditions viz. oxidative stress, inflammation, temperature and osmotic changes [68], [69], [70], [71], [72]. Several genes related to the components of MAPK signalling cascade (MAPKs as well as genes regulated by MAPK) including ERK1-ERK2 cascade (*mknk2a*, *mknk2b*, *fos*, *junbl*, *rps6ka1*, *egfra*) and MAPK signalling (*daxx*) were significantly induced during the transport process. MAPK pathway is triggered by a wide variety of stimuli, which in turn coordinately regulates a variety of cellular responses including activation of gene transcription, protein synthesis, ubiquitination and degradation, and immune responses [70], [73], [74]. Thus, the activation of genes in MAPK signalling pathways in liver could be pointing to their involvement in regaining homeostasis during the transport process.

### Immune Responses

Immune function in fish may be modulated when they encounter stress. Several immune-related genes were differentially regulated at different time points of the transport process similar to the observations in liver of several other fishes exposed to stress and during their recovery [32], [33], 75. Genes in feedback suppressors of cytokine signaling (SOCS) family regulates a large number of immune signaling pathways in mammals as well as in fish [76], [77]. After packing, *socs8* was induced, probably to regulate cytokine signaling that are necessary for normal homeostasis and cellular functions. Moreover, in another study on zebrafish, *sid4* was upregulated during viral infection [78]. This gene was also affected by the stress from the pre-transport procedures in the present study. On the other hand, several immune genes such as *c3b*, *tlr3* and *irf11* were inhibited at 48 and/or 72 h of transport. Thus, suppression of transcripts of the above-mentioned immune components in fish during transport could be attributed to the continued effect of cortisol resulting from various stressors including ammonia. The inhibitory effect of high ammonia on genes associated with innate immune response has been previously reported in zebrafish [13]. The suppression of transcripts of immune components during the transport may later on compromise the health of fish.

### Stress and Thyroid Hormone Regulation

An organism adapts to a stressful condition by regulating the circulating cortisol. In this context the enzyme 11β-hydroxysteroid dehydrogenase 2 (HSD11B2) plays a decisive role as it converts cortisol into inactive cortisone [79]. In the present study, whole body cortisol levels that increased immediately after packing were significantly decreased at 48 h of transport. This stress adjustment may be linked with the observed increase in the mRNA levels of *hsd11b2* at 0 h. The expression of *hsd11b2* in the kidney of zebrafish showed a time-delayed relationship with cortisol levels in a mock transport study [19] and upon application of a vortex stressor [80]. In addition to the genes that regulate cortisol levels, genes that control the thyroid hormone production were also examined here. Genes that code for *dio1* and *dio2* that are essential for peripheral regulation of thyroid hormone bioactivity [81] were downregulated during transport similar to the observations in Nile tilapia, *Oreochromis niloticus*, liver after dexamethasone injection [82]. It has been reported that thyroid hormones influence the stress response in fish by altering the functions of thyroid or stress hormones to regain homeostasis [83]. Inhibition of thyroid hormone transcripts associated with the transport process may imply their contribution to stress adaptation.

### Conclusions

Transcriptome analysis demonstrated that genes related to a variety of biological processes and pathways were regulated in the liver of zebrafish that were subjected to pre-transport procedures and transport itself. Most of the adaptive responses were observed during transport when the fish experienced extended stress from the collective effect of various chemical and physical stressors. Transcriptional changes of genes linked to gluconeogenesis and glycolysis may be indicating an increased dependence on glucose for energy during fish transport. Inhibition of genes linked to fatty acid beta-oxidation may be pointing to the fisheś poor ability to utilize energy from fatty acids. Even though the genes that code for some transaminases were inhibited upon high ammonia exposure, sustained glutamate deamination may have led to the high ammonia accumulation in liver/body, which in turn probably activates the ammonia detoxification mechanisms. Alterations in transcripts related to ubiquitination, ROS generation, MAPK signalling pathway may be denoting the importance of cellular and tissue responses in fish during the transport process. A negative impact on the health of fish could also be interpreted from the suppression of the transcripts of some of the immune components. Although transcriptome analyses are useful indicators of physiology, the consequence of gene alterations can be validated only through the direct measurements of their end products. Therefore, the conclusions drawn from changes in transcriptome are only indicative of their physiological consequences. Further follow up studies on the adaptive mechanisms identified in this study are therefore warranted. Such efforts are needed to develop strategies for improving the welfare of shipped fish.

## Supporting Information

File S1 Supporting tables. Table S1 Primers used for quantitative real time PCR. Table S2 Water quality parameters measured in the transport bags during mock transport of zebrafish. Table S3 Full list of genes that are significantly upregulated in the zebrafish liver during the transport process. Table S4 Full list of genes that are significantly downregulated in the zebrafish liver during the transport process.
(PDF)
