# Supplementary material for: Liver Transcriptome Changes in Zebrafish during Acclimation to Transport-Associated Stress
Source: PLoS One. 2013 Jun 10;8(6):e65028. doi: 10.1371/journal.pone.0065028 (PMC3677916; doi:10.1371/journal.pone.0065028)
Supplement: File S1 — Supporting tables. Table S1 Primers used for quantitative real time PCR. Table S2 Water quality parameters measured in the transport bags during mock transport of zebrafish. Table S3 Full list of genes that are significantly upregulated in the zebrafish liver during the transport process. Table S4 Full list of genes that are significantly downregulated in the zebrafish liver during the transport process. (PDF) [file pone.0065028.s001.pdf]

Table S1 Primers used for quantitative real time PCR

Gene	Primer sequence (5' to 3')	Size (bp)	E (%)	GenBank accession no:
scd [a]	F: ACCCGGAAGTCATCGAGAGA	188	99.1	NM_198815
	R: GAGGAGCGTCGGGATGAAAT			
got2a [a]	F: GTCTTACAGCTCCGGGCAAG	170	98.0	NM_213379
	R: ACACTGCTGAGGACGAATGG			
ube2h [a]	F: GGACCACAAGGAACACCGTA	147	99.4	NM_201489
	R: TAAACACACGGTCCCTGACG			
gpx4b [a]	F: GAGGTACGGACCAATGGACG	78	99.7	NM_001030070
	R: GCCGCAGCATACACACTCTA			
mknk2b [a]	F: ACACATTTAGCCCCTACCGC	184	99.3	NM_194402
	R: GCAACAACGGTGGTCTTAGC			
hsd11b2 [b]	F: TGCTGCTGGCTGTACTTCAC	123	99.5	NM_212720.2
	R: TGCATCCAACTTCTTTGCTG			
eef1a [c]	F: GAGGAAATCACCAAGGAAGTCAG	134	99.0	NM131263.1
	R: TTGAACCAGCCCATGTTTGAG			
b2m [d]	F: GCCTTCACCCCAGAGAAAGG	101	99.8	BC062841
	R: GCGGTTGGGATTTACATGTTG			

E-amplification efficiency

scd- stearoyl-CoA desaturase (delta-9-desaturase), got2a- glutamic-oxaloacetic transaminase 2, ube2h- ubiquitin-conjugating enzyme E2H, gpx4b- glutathione peroxidase 4b, mknk2b- MAP

kinase-interacting serine/threonine kinase 2b, *hsd11b2- 11β-hydroxysteroid dehydrogenase 2*, *eef1a-elongation factor 1-alpha* and *b2m-beta-2-microglobulin*.

Primers designed- [a] during the present study, [b] [1], [c] [2] and [d] [3].

1. Alsop D, Vijayan MM (2008) Development of the corticosteroid stress axis and receptor expression in zebrafish. Am J Physiol Regul Integr Comp Physiol 294: R711-R719.
2. Sundaram A, Consuegra S, Kiron V, Fernandes J (2012) Positive selection pressure within teleost toll-like receptors *tlr21* and *tlr22* subfamilies and their response to temperature stress and microbial components in zebrafish. Mol Biol Rep. doi:10.1007/s11033-012-1765-y.
3. McCurley AT, Callard GV (2008) Characterization of housekeeping genes in zebrafish: male-female differences and effects of tissue type, developmental stage and chemical treatment. BMC Mol Biol 9: 102.

Additional file 2: Table S2 Water quality parameters measured in the transport bags during mock transport of zebrafish

Water parameter	Time point		
	0 h	48 h	72 h
TAN (mg l^{-1})	0.10 ± 0.01	18.13 ± 1.25	32.77 ± 0.25
Nitrite (mg l^{-1})	0.008 ± 0.002	0.04 ± 0.003	0.039 ± 0.004
Nitrate (mg l^{-1})	4. 31 ± 0.12	6.77 ± 0.69	9. 69 ± 0.95
pH	7.67 ± 0.08	6.82 ± 0.08	6.55 ± 0.19
Oxygen saturation (%)	130.5 ± 2.1	203.3 ± 9.94	191.3 ± 13.38
Temperature (°C)	24.2 ± 0.048	24.5 ± 0.087	24.3 ± 0.041

The average of the measured water quality parameters from the four randomly selected transport units from which fish were collected for studying the physiological markers and microarray analysis, immediately after packing (0 h), and at 48 and 72 h during transport of zebrafish are indicated. Values are given as means ± s.e.m..

Additional file 3: Table S3 Full list of genes that are significantly upregulated in the zebrafish liver during transport process.

Only after packing (0 h)

Gene symbol	Gene name	Fold change*		
		0 h	48 h	72 h
a2ml	*alpha-2-macroglobulin-like [NM_001139479]*	1,37	-1,62	-1,90
aldh6a1	*aldehyde dehydrogenase 6 family, member A1 [NM_001002374]*	1,32	-1,42	-1,52
aldob	*aldolase b, fructose-bisphosphate [NM_194367]*	2,27	1,29	1,17
asah1b	*N-acylsphingosine amidohydrolase (acid ceramidase) 1b [NM_200577]*	1,58	-1,73	-1,77
atp6v0cb	*ATPase, H+ transporting, lysosomal, V0 subunit c, b [NM_205554]*	2,24	1,66	1,62
bactin2	*bactin2 [NM_181601]*	2,03	1,10	1,19
cel.1	*carboxyl ester lipase, tandem duplicate 1 [NM_199607]*	1,38	-1,29	-2,44
ces2	*carboxylesterase 2 (intestine, liver) [NM_001077252]*	1,78	-1,89	-1,77
cmbl	*carboxymethylenebutenolidase-like (Pseudomonas) [NM_001109832]*	2,21	-1,52	1,25
cox4i2	*cytochrome c oxidase subunit IV isoform 2 [NM_200803]*	2,88	-1,10	-1,30
crbn	*cereblon [BC080253]*	1,67	-1,46	-1,74
cry1a	*cryptochrome 1a [NM_001077297]*	2,17	-2,38	-2,89
cry2a	*cryptochrome 2a [NM_131791]*	2,17	1,02	1,40
cry5	*cryptochrome 5 [NM_131788]*	2,12	-1,11	1,11
cyp2k22	*cytochrome P450, family 2, subfamily K, polypeptide 22 [NM_200235]*	3,54	1,62	1,63
cyp2x12	*cytochrome P450 CYP2X12 [NM_001079853]*	1,88	-1,31	-1,05
cyp8b2	*cytochrome P450, family 8, subfamily B, polypeptide 2 [NM_001110288]*	2,03	-1,11	1,26
dcbld1	*discoidin, CUB and LCCL domain containing 1 [NM_001039977]*	3,15	-3,35	-4,33
dio1	*deiodinase, iodothyronine, type I [NM_001007283]*	1,54	-2,24	-1,85
egr1	*early growth response 1 [NM_131248]*	6,01	2,69	1,24
ehf	*ets homologous factor [NM_001039822]*	1,77	1,02	-1,18
eno1	*enolase 1, (alpha) [NM_212722]*	1,83	1,12	-1,11
ets2	*v-ets erythroblastosis virus E26 oncogene homolog 2 (avian) [NM_001023580]*	2,62	1,16	-1,14

fam53b	*family with sequence similarity 53, member B [NM_001007187]*	2,06	1,55	1,15
foxa1	*forkhead box A1 [NM_131284]*	1,37	-1,52	-1,23
ft1	*alpha(1,3)fucosyltransferase gene 1 [NM_131409]*	3,69	1,07	1,44
gch1	*GTP cyclohydrolase 1 [NM_001136255]*	7,53	2,96	1,82
hpx	*hemopexin [NM_001111147]*	3,05	1,05	1,02
hsd11b2	*hydroxysteroid 11-beta dehydrogenase 2 [NM_212720]*	3,95	1,19	2,10
igf2bp2a	*insulin-like growth factor 2 mRNA binding protein 2a [NM_001114558]*	1,14	1,92	2,64
im:7159406	*im:7159406 [CK694681]*	1,20	-1,85	-1,18
itih3	*inter-alpha (globulin) inhibitor H3 [BC097018]*	2,42	-1,11	-1,17
kazald2	*Kazal-type serine peptidase inhibitor domain 2 [NM_001105124]*	1,52	-1,78	-2,21
LOC100001210	*hypothetical protein LOC100001210 [NM_001114695]*	3,33	1,75	2,00
LOC100007703	*hypothetical protein LOC100007703 [NM_001111252]*	3,01	-1,28	1,50
LOC100302390	*hypothetical protein LOC100302390 [NM_001163005]*	2,01	-1,20	-1,18
LOC100334215	*LOC100334215 [BC150430]*	1,64	-1,18	-1,64
LOC555748	*UDP-glucose pyrophosphorylase 2 [NM_001145557]*	1,78	-1,46	-1,05
LOC796447	*hypothetical protein LOC796447 [NM_001100056]*	2,02	-1,58	-2,08
LOC799067	*LOC799067 [BC127401]*	1,79	-1,15	1,64
mao	*monoamine oxidase [NM_212827]*	1,33	-2,34	-1,43
mdh1a	*malate dehydrogenase 1a, NAD (soluble) [NM_199947]*	1,84	-1,07	-1,09
mfsd4b	*major facilitator superfamily domain containing 4b [NM_001114416]*	3,17	-1,22	-1,30
mov10b.2	*moloney leukemia virus 10, homolog, b.2 [NM_001044340]*	2,44	1,04	1,81
ms4a17a.6	*membrane-spanning 4-domains, subfamily A, member 17A.6 [NM_001083012]*	2,06	1,01	1,03
nav3	*neuron navigator 3 [NM_001045143]*	3,56	2,00	1,32
ncor1	*nuclear receptor co-repressor 1 [NM_200276]*	1,29	-2,21	-1,54
neurla	*neuralized homolog a (Drosophila) [NM_001077558]*	3,35	2,19	2,01
nfia	*nuclear factor I/A [NM_001079962]*	1,24	-1,68	-1,75
nxnl1	*novel protein similar to vertebrate thioredoxin-like 6 (TXNL6) [NM_001110760]*	1,82	-1,34	-1,21
oatx	*organic anion transporter X*	5,90	-1,95	1,20
oxct1b	*3-oxoacid CoA transferase 1b [NM_001077150]*	1,39	-1,95	-1,44
pacsin3	*protein kinase C and casein kinase substrate in neurons 3 [NM_200989]*	1,66	-1,22	-1,02

per2	*period 2 [FJ435338]*	2,39	1,16	1,10
pgk1	*phosphoglycerate kinase 1 [NM_213387]*	1,47	-1,36	-1,32
phyh	*phytanoyl-CoA 2-hydroxylase [NM_001017823]*	1,73	-1,57	-1,21
pklr	*pyruvate kinase, liver and RBC [NM_201289]*	1,69	-2,00	-3,33
rhobtb2a	*Rho-related BTB domain containing 2a [NM_001099974]*	1,94	-1,30	-1,25
roraa	*RAR-related orphan receptor A, paralog a [NM_001110167]*	2,60	1,30	1,48
sb:cb120	*sb:cb120 [CN015641]*	1,77	1,06	-1,14
serpinf1	*serine (or cysteine) peptidase inhibitor, clade F, member 1 [NM_001004539]*	1,88	-1,89	-1,94
sesn1	*sestrin 1 [NM_001002660]*	1,99	-1,34	1,09
shbg	*sex hormone binding globulin [NM_001007151]*	1,79	-1,35	-1,15
si:ch1073-440b2.1	*si:ch1073-440b2.1 [AI384705]*	1,58	-1,28	-1,07
si:ch211-215l11.5	*si:ch211-215l11.5 [NM_001163806]*	4,60	1,89	1,52
si:ch211-220f13.1	*si:ch211-215l11.5 [NM_001163806]*	4,60	1,89	1,52
si:ch211-240l19.5	*si:ch211-240l19.5 [NM_001030152]*	1,64	1,17	-1,92
si:ch211-284e20.8	*si:ch211-284e20.8 [NM_001099265]*	1,24	-2,27	-1,57
si:ch73-252g14.4	*si:ch73-252g14.4 [NM_001100029]*	2,03	-1,88	-3,24
si:dkey-39a18.1	*si:dkey-39a18.1 [NM_001025494]*	2,61	-1,36	-1,08
si:dkey-91i10.3	*si:dkey-91i10.3 [NM_001123277]*	2,16	1,19	1,64
si:dkey-37m8.9	*si:dkey-37m8.9 [XM_686251]*	1,93	-2,56	-2,06
sid4	*secreted immunoglobulin domain 4 [NM_001034182]*	1,72	-1,36	-1,31
slc1a4	*solute carrier family 1 (glutamate/neutral amino acid transporter), member 4 [NM_001002513]*	4,08	-1,64	-1,93
slc25a47a	*solute carrier family 25, member 47a [NM_001045314]*	3,91	-1,90	-1,81
slc27a2	*solute carrier family 27 (fatty acid transporter), member 2 [NM_001025299]*	1,67	-1,37	-1,21
socs8	*suppressor of cytokine signaling 8 [NM_001114554]*	2,08	-1,49	1,23
sort1b	*sortilin 1b [NM_001126465]*	1,47	-1,60	-1,56
st6galnac4	*ST6 (alpha-N-acetyl-neuraminyl-2,3-beta-galactosyl-1,3)-N-acetylgalactosaminide alpha-2,6-sialyltransferase 4 [NM_001045370]*	2,48	1,15	1,11
sult2st1	*sulfotransferase family 2, cytosolic sulfotransferase 1 [NM_198914]*	1,82	-1,18	-1,04
tef	*thyrotroph embryonic factor [NM_131400]*	2,10	-2,64	-2,44

tfa	*transferrin-a [NM_001015057]*	1,64	-2,19	-1,73
tfr1a	*transferrin receptor 1a [NM_001009917]*	2,20	-1,24	1,06
tgfbi	*transforming growth factor, beta-induced [NM_182862]*	1,04	-2,48	-2,21
tmem88a	*transmembrane protein 88 a [NM_205670]*	1,80	-1,39	-1,15
tmem88b	*transmembrane protein 88 b [NM_001077144]*	1,21	-1,97	-1,84
tp53inp1	*tumor protein p53 inducible nuclear protein 1 [NM_001080036]*	3,80	1,69	1,79
tspan13b	*tetraspanin 13b [NM_001005970]*	1,94	1,27	-1,23
tuba8l2	*tubulin, alpha 8 like 2 [NM_200691]*	2,10	-1,34	-1,84
uckl1	*uridine-cytidine kinase 1-like 1 [NM_001128259]*	3,05	1,01	-1,33
ugt5b6	*UDP glucuronosyltransferase 5 family, polypeptide B6 [NM_001040345]*	1,64	-2,07	-1,39
uox	*urate oxidase [NM_001002332]*	1,63	-2,06	-1,82
upb1	*ureidopropionase, beta [NM_199616]*	1,48	-2,26	-2,07
wif1	*wnt inhibitory factor 1 [NM_131229]*	2,29	-1,03	-1,02
wu:fb39d01	*wu:fb39d01 [CN500264]*	2,22	1,12	1,78
wu:fb51f10	*wu:fb51f10 [CN319073]*	2,60	1,27	1,56
wu:fc64h08	*wu:fc64h08 [AI878716]*	2,28	-1,21	-1,12
wu:fd10h03	*wu:fd10h03 [AW018998]*	1,95	-1,06	-1,07
xpnpep1	*X-prolyl aminopeptidase (aminopeptidase P) 1, soluble [NM_212980]*	1,80	-1,10	-1,26
zfp36l1b	*zinc finger protein 36, C3H type-like 1b [NM_199649]*	3,95	1,02	-1,01
zgc:100846	*zgc:100846 [NM_205603]*	2,50	1,40	1,42
zgc:101761	*zgc:101761 [NM_001007773]*	2,11	1,38	1,31
zgc:103586	*zgc:103586[NM_001007389]*	1,69	-1,70	-1,01
zgc:112368	*zgc:112368 [NM_001025474]*	2,01	-1,04	-1,85
zgc:113456	*zgc:113456 [NM_001030072]*	2,47	-1,49	-1,23
zgc:123258	*zgc:123258 [NM_001037439]*	2,00	-1,10	-1,18
zgc:123295	*zgc:123295 [NM_001037562]*	2,29	1,47	-1,35
zgc:153913	*zgc:153913 [NM_001079979]*	1,88	-1,22	1,03
zgc:162396	*zgc:162396 [NM_001044782]*	1,14	-1,90	-1,84
zgc:171489	*zgc:171489 [NM_001122620]*	2,10	1,08	1,56
zgc:171674	*zgc:171674 [NM_001105106]*	1,67	-1,27	-1,03

zgc:56136	*zgc:56136 [NM_200198]*	1,93	-1,53	-1,96
zgc:65811	*zgc:65811 [NM_200552]*	2,52	-1,23	1,12
zgc:65857	*zgc:65857 [NM_200580]*	1,80	-1,30	-1,25
zgc:66382	*zgc:66382 [NM_199605]*	1,29	-1,22	-1,97
zgc:77778	*zgc:77778 [NM_213203]*	2,23	-2,00	-2,93
zgc:92041	*zgc:92041 [NM_001003737]*	2,19	-1,00	-2,83
zgc:92046	*zgc:92046 [NM_001002389]*	2,98	1,68	1,79
zgc:92745	*zgc:92745 [NM_001003426]*	2,66	-1,04	-4,73

only during transport (at 48 and/or 72 h)

**Gene symbol**	**Gene name**	**Fold change***		
		**0 h**	**48 h**	**72 h**
acap3b	*ArfGAP with coiled-coil, ankyrin repeat and PH domains 3b [NM_001083015]*	-1,27	1,86	1,82
accn2a	*amiloride-sensitive cation channel 2 a [NM_214791]*	1,93	2,59	2,10
acer1	*alkaline ceramidase 1 [NM_001017603]*	-1,20	3,32	2,25
aclya	*ATP citrate lyase a [NM_001002649]*	1,80	4,08	3,93
acot11a	*acyl-CoA thioesterase 11a [NM_001099975]*	1,83	3,00	4,04
adipor1a	*adiponectin receptor 1a [NM_001002467]*	1,33	2,60	1,98
agfg1a	*ArfGAP with FG repeats 1a [NM_199835]*	1,64	3,48	3,17
and4	*actinodin4 [NM_001136244]*	1,01	1,25	3,12
ankrd13a	*ankyrin repeat domain 13A[NM_001126387]*	1,47	2,01	2,13
anp32b	*acidic (leucine-rich) nuclear phosphoprotein 32 family, member B [AI964260]*	1,21	-1,10	1,92
arhgap28	*Rho GTPase activating protein 28 [NM_001007435]*	-1,12	3,19	2,56
arid3b	*AT rich interactive domain 3B (Bright like) [NM_001083002]*	2,00	2,93	3,75
arl3	*ADP-ribosylation factor-like 3 [NM_001044908]*	-1,11	1,81	1,57
arl4ab	*ADP-ribosylation factor-like 4ab [NM_200318]*	2,16	2,85	1,94
atad1a	*ATPase family, AAA domain containing 1a [NM_001007112]*	-1,20	1,92	2,01
atf7ip	*activating transcription factor 7 interacting protein [NM_001077603]*	-1,28	-1,11	1,57

bdnf	*brain-derived neurotrophic factor [NM_131595]*	1,60	1,38	2,50
bmi1	*B lymphoma Mo-MLV insertion region 1 [NM_194366]*	1,21	2,12	1,79
bmp2k	*BMP2 inducible kinase [NM_001008644]*	-1,24	1,46	2,02
bnip3	*BCL2/adenovirus E1B interacting protein 3 [NM_001012245]*	1,60	1,85	2,16
brp44	*brain protein 44 [NM_212592]*	1,84	5,85	7,19
btbd2	*BTB (POZ) domain containing 2 [NM_001045092]*	1,34	1,90	2,72
btbd6a	*BTB (POZ) domain containing 6a [NM_001114925]*	1,14	-2,00	1,31
btg2	*B-cell translocation gene 2 [NM_130922]*	1,00	2,65	1,98
bzw1b	*basic leucine zipper and W2 domains 1b [NM_213092]*	1,68	3,59	2,14
calub	*calumenin b [NM_201082]*	1,10	2,19	1,67
cant1b	*calcium activated nucleotidase 1b [NM_001003595]*	1,59	1,60	2,64
cbx2	*chromobox homolog 2 (Drosophila Pc class) [NM_194373]*	1,03	2,84	2,71
cbx4	*chromobox homolog 4 (Pc class homolog, Drosophila) [NM_205749]*	-1,11	3,12	3,43
ccdc120	*coiled-coil domain containing 120 [NM_001077451]*	1,58	1,38	2,71
ccnd3	*cyclin D3 [NM_001006017]*	1,51	1,82	2,19
cd164	*CD164 molecule, sialomucin [NM_001044791]*	1,39	1,96	3,32
cdc34a	*cell division cycle 34 homolog (S. cerevisiae) a [NM_200958]*	-1,16	1,74	1,75
cdc7	*cell division cycle 7 homolog (S. cerevisiae) [NM_001007409]*	-1,28	2,36	2,38
cebp1	*CCAAT/enhancer binding protein (C/EBP) 1 [NM_131837]*	1,46	1,48	2,53
cep76	*centrosomal protein 76 [NM_001103119]*	1,30	2,43	2,64
cited3	*Cbp/p300-interacting transactivator, with Glu/Asp-rich carboxy-terminal domain, 3 [NM_200078]*	1,45	2,15	1,81
cldn7b	*claudin 7b [NM_131637]*	-1,33	2,58	1,88
clic4	*chloride intracellular channel 4 [NM_201486]*	-1,31	1,57	1,76
cln8	*ceroid-lipofuscinosis, neuronal 8 [NM_001114588]*	1,04	3,83	2,67
clock	*clock [NM_130957]*	2,05	2,37	3,45
cnnm2a	*cyclin M2a [NM_001144785]*	1,07	1,87	2,58
col4a3bpa	*collagen, type IV, alpha 3 (Goodpasture antigen) binding protein a [NM_001100960]*	1,34	2,43	1,93
crlf1a	*cytokine receptor-like factor 1a [NM_001002650]*	1,52	1,54	2,45

csnk1da	*casein kinase 1, delta a [NM_199583]*	-1,02	2,10	1,39
csrnp1b	*cysteine-serine-rich nuclear protein 1b [NM_199619]*	2,39	3,14	2,64
ctbp2	*C-terminal binding protein 2 [NM_001195491]*	1,05	1,68	2,82
ctdspl2b	*CTD (carboxy-terminal domain, RNA polymerase II, polypeptide A)*			
	small phosphatase like 2b [NM_001089326]	-1,12	1,70	1,83
ctnna2	*catenin (cadherin-associated protein), alpha 2 [NM_001159372]*	1,12	2,11	1,43
ctnnbip1	*catenin, beta interacting protein 1 [NM_131594]*	-1,22	1,83	1,55
cul4b	*cullin 4B [NM_001122844]*	1,33	1,44	2,03
cyfip2	*cytoplasmic FMR1 interacting protein 2 [NM_001097587]*	1,81	2,29	2,27
cyp11a1	*cytochrome P450, subfamily XIA, polypeptide 1 [NM_152953]*	-2,39	2,01	1,42
cyp2k18	*cytochrome P450, family 2, subfamily K, polypeptide 18 [NM_200512]*	1,95	1,30	3,08
dachd	*dachshund d [NM_001080673]*	1,49	1,73	2,25
daxx	*death-associated protein 6 [NM_001044949]*	1,14	2,33	1,93
dazl	*daz-like gene [NM_131524]*	-1,18	2,68	1,99
dbx1b	*developing brain homeobox 1b [NM_131178]*	-1,21	2,92	4,06
dbx2	*homeobox protein [AF030286]*	2,35	1,10	3,09
ddx21	*DEAD (Asp-Glu-Ala-Asp) box polypeptide 21 [NM_001127335]*	1,60	2,62	2,57
ddx5	*DEAD (Asp-Glu-Ala-Asp) box polypeptide 5 [NM_212612]*	1,24	2,95	2,63
dgat1a	*diacylglycerol O-acyltransferase homolog 1a (mouse) [NM_199730]*	-1,04	1,74	2,36
dlat	*dihydrolipoamide S-acetyltransferase*			
	(E2 component of pyruvate dehydrogenase complex) [NM_212667]	1,39	2,24	1,78
dnajb1b	*DnaJ (Hsp40) homolog, subfamily B, member 1b [NM_199773]*	-1,00	2,32	1,71
dnajb6b	*DnaJ (Hsp40) homolog, subfamily B, member 6b [NM_200305]*	1,06	2,04	1,72
dnd	*dead end [NM_212795]*	-1,29	2,35	1,84
dusp22a	*dual specificity phosphatase 22a [NM_001002514]*	1,19	2,32	1,86
dyrk1b	*dual-specificity tyrosine-(Y)-phosphorylation regulated kinase 1B [NM_001168265]*	1,91	1,66	3,11
elovl4b	*elongation of very long chain fatty acids*			
	(FEN1/Elo2, SUR4/Elo3, yeast)-like 4b [NM_199972]	-1,11	1,66	2,09
enpp1	*ectonucleotide pyrophosphatase/phosphodiesterase 1 [NM_001030168]*	1,40	2,26	3,05
entpd4	*ectonucleoside triphosphate diphosphohydrolase 4 [NM_001002419]*	-1,52	1,48	1,10

epb41l5	*erythrocyte membrane protein band 4.1 like 5 [NM_200089]*	1,51	2,79	2,11
epha4a	*eph receptor A4a [NM_001005919]*	1,60	1,25	2,32
fa2h	*fatty acid 2-hydroxylase [NM_001045420]*	-1,23	1,11	2,08
fam46ba	*family with sequence similarity 46, member Ba [NM_001077806]*	2,00	2,69	1,16
fancg	*Fanconi anemia, complementation group G [NM_205639]*	-1,68	1,80	1,61
far1	*fatty acyl CoA reductase 1 [NM_213508]*	1,68	2,41	2,40
fgd6	*FYVE, RhoGEF and PH domain containing 6 [NM_001099980]*	-1,03	1,57	2,06
fgf8a	*fibroblast growth factor 8 a [NM_131281]*	-1,61	1,89	3,71
ficd	*FIC domain containing [NM_001017794]*	-1,94	1,58	1,47
foxe3	*forkhead box E3 [NM_001079682]*	1,26	1,25	2,25
g3bp2	*GTPase activating protein (SH3 domain) binding protein 2 [NM_001080563]*	1,19	2,04	1,44
gdf6a	*growth differentiation factor 6a [NM_001159994]*	1,09	3,01	2,66
grtp1a	*growth hormone regulated TBC protein 1a [NM_213402]*	-1,05	2,17	2,11
grtp1b	*growth hormone regulated TBC protein 1b [NM_001017810]*	1,13	3,76	3,47
gsnb	*gelsolin b [NM_001089344]*	1,04	3,14	3,36
gss	*glutathione synthetase NM_001006104]*	-1,24	1,69	1,43
hbegfa	*heparin-binding EGF-like growth factor a [NM_001111226]*	-1,15	1,88	1,27
hiat1b	*hippocampus abundant transcript 1b [NM_213527]*	-1,40	1,61	1,28
hic2	*hypermethylated in cancer 2 [NM_182869]*	1,34	1,37	2,08
hif1al	*hypoxia-inducible factor 1, alpha subunit, like [NM_200405]*	2,17	4,80	4,07
hmga1a	*high mobility group AT-hook 1a [NM_213168]*	-1,17	1,25	1,72
hmga2	*high mobility group AT-hook 2 [NM_212680]*	1,62	-2,00	1,64
hoxb1b	*homeo box B1b [NM_131142]*	1,12	1,70	2,34
hoxc5a	*homeo box C5a [NM_131144]*	1,20	2,06	2,75
hoxc6a	*homeo box C6a [NM_131123]*	2,39	3,60	5,88
hoxc8a	*homeo box C8a [NM_001005771]*	1,90	1,11	3,41
hoxc9a	*homeo box C9a [NM_131528]*	1,80	3,93	5,49
hoxd12a	*homeo box D12a [NM_001126486]*	-1,08	2,84	1,71
hps1	*Hermansky-Pudlak syndrome 1 [NM_001037688]*	-1,14	2,15	2,00
hsd17b1	*hydroxysteroid (17-beta) dehydrogenase 1[NM_205584]*	-1,32	1,64	1,33

hsd17b3	*hydroxysteroid (17-beta) dehydrogenase 3 [NM_200364]*	7,81	12,46	36,63
hsh2d	*hematopoietic SH2 domain containing [NM_001017541]*	1,41	2,53	2,47
hsp70	*heat shock cognate 70-kd protein [NM_131397]*	1,49	3,30	2,62
hsp70l	*heat shock cognate 70-kd protein, like [NM_001113589]*	1,83	3,46	3,74
hspa4a	*heat shock protein 4a [NM_214716]*	-1,26	1,65	1,40
igf2bp2a	*insulin-like growth factor 2 mRNA binding protein 2a [NM_001114558]*	1,14	1,92	2,64
igfbp1b	*insulin-like growth factor binding protein 1b [NM_001098257]*	2,87	9,11	6,80
igfbp3	*insulin-like growth factor binding protein 3 [NM_205751]*	3,15	6,47	4,47
ikbke	*inhibitor of kappa light polypeptide gene enhancer in B-cells, kinase epsilon [NM_001002751]*	-1,03	1,29	2,05
il23r	*interleukin 23 receptor [NM_001113506]*	1,87	3,09	3,53
im:7138933	*im:7138933 [CK677939]*	1,23	1,56	2,01
inadl	*InaD-like (Drosophila) [NM_001127185]*	-1,08	1,54	2,07
inpp5f	*inositol polyphosphate-5-phosphatase F [NM_001126432]*	1,09	2,62	2,28
ipmkb	*inositol polyphosphate multikinase b [NM_001080064]*	1,12	2,02	1,55
isl1l	*islet1, like [NM_001002043]*	1,34	3,41	1,69
isoc1	*isochorismatase domain containing 1 [NM_001079981]*	1,08	1,55	1,78
ivns1abpb	*influenza virus NS1A binding protein b [NM_201483]*	-1,23	2,16	1,70
kirrelb	*kin of IRRE like b [NM_001130651]*	1,98	-1,02	2,20
klc1	*kinesin light chain 1 [NM_199954]*	1,73	1,20	2,48
klc3	*kinesin light chain 3 [NM_001077178]*	1,14	2,19	2,19
klhl24	*kelch-like 24 (Drosophila) [NM_001077340]*	1,04	2,38	2,67
kras	*v-Ki-ras2 Kirsten rat sarcoma viral oncogene homolog [NM_001003744]*	1,14	-1,41	3,81
lamp2	*lysosomal membrane glycoprotein 2[NM_001013533]*	-1,01	2,19	1,62
large	*like-glycosyltransferase [NM_001004537]*	1,13	4,09	3,18
ldhba	*lactate dehydrogenase Ba [NM_131247]*	1,40	2,14	2,30
LOC100000565	*hypothetical LOC100000565 [BC116590]*	1,26	-1,07	1,97
LOC100002400	*caldesmon 1-like [XM_001338635]*	-1,10	2,10	1,56
LOC100005476	*FXYD domain containing ion transport regulator 7-like [NM_001201426]*	-1,11	2,67	1,99
LOC100150511	*RGM domain family, member B-like [NM_001200057]*	1,11	1,53	2,24

LOC100329706	*ATP-binding cassette sub-family C member 8-like [XR_117895]*	1,35	5,86	3,67
LOC100331916	*dynamin-1-like [BC142800]*	-1,26	1,72	2,69
LOC557375	*similar to cold shock domain containing C2, RNA binding [XR_084527]*	1,04	1,94	2,19
LOC559239	*hypothetical LOC559239 [BC090446]*	-1,40	2,49	2,33
LOC560232	*hypothetical LOC560232 [NM_001123254]*	1,96	1,27	3,18
LOC562098	*hypothetical LOC562098 [XM_701777]*	-1,09	2,03	2,10
LOC563777	*similar to Proteasome-associated protein ECM29 homolog [NM_001162486]*	1,31	3,77	4,09
LOC564868	*hypothetical LOC564868 [NM_001111180]*	4,52	11,18	10,59
LOC565175	*hypothetical LOC565175 [NM_001089407]*	-1,02	2,36	2,54
LOC567504	*interferon-induced protein with tetratricopeptide repeats 5-like [NM_001190465]*	1,84	2,74	2,31
LOC571530	*hypothetical LOC571530 [XM_695132]*	1,31	2,98	3,18
LOC792383	*hypothetical protein LOC792383 [BC153946]*	1,64	2,39	2,72
lrrc8a	*leucine rich repeat containing 8 family, member A [NM_001029949]*	1,15	2,13	1,65
ltk	*leukocyte tyrosine kinase [NM_001006660]*	1,24	1,81	2,27
mab21l1	*mab-21-like 1 [NM_152974]*	1,44	-1,66	2,60
mast3	*microtubule associated serine/threonine kinase 3 [CV119686]*	-1,21	1,53	1,67
mcm10	*minichromosome maintenance complex component 10 (mcm10) [NM_001024813]*	-1,51	2,31	1,60
me1	*malic enzyme 1, NADP(+)-dependent, cytosolic [NM_001077338]*	1,47	8,70	10,07
med24	*mediator complex subunit 24 [NM_001039062]*	-1,04	1,99	1,55
mef2ca	*myocyte enhancer factor 2ca [NM_131312]*	1,44	1,40	2,08
mepce	*methylphosphate capping enzyme [NM_001128529]*	1,39	1,39	2,08
mfsd6a	*major facilitator superfamily domain containing 6a [NM_001083056]*	1,49	2,19	1,94
mgat5	*mannoside acetylglucosaminyltransferase 5 [NM_001045311]*	-1,25	1,41	1,63
mknk2a	*MAP kinase-interacting serine/threonine kinase 2a [NM_212723]*	1,40	2,66	3,41
mmp9	*matrix metalloproteinase 9 [NM_213123]*	1,27	4,76	2,41
mon1a	*MON1 homolog A (yeast) [NM_001098770]*	2,29	2,98	6,69
mpp6b	*membrane protein, palmitoylated 6b (MAGUK p55 subfamily member 6) [NM_001044777]*	1,50	2,06	2,67
mppe1	*metallophosphoesterase 1 [NM_001017701]*	-1,20	2,18	1,55
mtmr10	*myotubularin related protein 10 [NM_001077597]*	-1,46	1,27	1,71

mtmr12	*myotubularin related protein 12 [NM_001113613]*	1,08	2,48	2,84
mtmr1a	*myotubularin related protein 1a [NM_001020721]*	1,24	2,56	2,35
mtmr7a	*myotubularin related protein 7a [NM_001040316]*	1,08	4,61	3,75
nap1l4b	*nucleosome assembly protein 1-like 4b [NM_001007453]*	1,54	2,62	1,68
nbea	*neurobeachin [NM_001013500]*	-1,05	1,54	2,03
ncs1a	*neuronal calcium sensor 1a [NM_200465]*	1,37	3,79	5,01
nfasca	*neurofascin homolog (chicken) a [NM_001145767]*	1,61	1,93	2,69
nfya	*nuclear transcription factor Y, alpha [NM_001082795]*	1,38	2,36	1,74
nkain4	*Na+/K+ transporting ATPase interacting 4 [NM_001113646]*	1,37	1,99	2,84
nkx6.3	*NK6 homeobox 3 [NM_001166140]*	1,07	2,54	4,23
nog1	*noggin 1 [NM_130983]*	1,00	1,22	2,20
nog3	*noggin 3 [NM_130982]*	1,30	2,10	7,72
npsn	*nephrosin [NM_205756]*	1,53	4,19	3,09
nptna	*neuroplastin a [NM_001160156]*	1,13	1,26	2,20
nrarpb	*notch-regulated ankyrin repeat protein b [NM_181496]*	-1,01	1,97	2,20
nrg1	*neuregulin 1 [NM_001044911]*	1,23	2,27	3,05
nrxn1a	*neurexin 1a [NM_001080021]*	1,01	-1,53	2,13
nusap1	*nucleolar and spindle associated protein 1 [NM_001145694]*	-1,35	2,64	1,79
nxph1	*neurexophilin 1 [NM_001002733]*	-3,45	1,81	1,92
otomp	*otolith matrix protein [NM_001045087]*	1,20	1,96	2,87
ovol1	*ovo-like 1 (Drosophila) [NM_001114744]*	-1,51	1,40	1,48
pabpc4	*poly(A) binding protein, cytoplasmic 4 (inducible form) [NM_201296]*	1,56	2,05	1,82
pacs1	*phosphofurin acidic cluster sorting protein 1 [NM_001098748]*	-1,06	2,21	1,83
pacs2	*phosphofurin acidic cluster sorting protein 2 [NM_001122751]*	-1,45	1,60	2,13
pald	*paladin [NM_198811]*	-1,01	2,11	1,73
pcbp2	*poly(rC) binding protein 2 [NM_201192]*	1,17	2,34	2,99
pcdh19	*protocadherin 19 [NM_001127519]*	-1,17	1,03	2,77
pcgf5b	*polycomb group ring finger 5b [NM_001045235]*	1,20	2,11	2,44
pck2	*phosphoenolpyruvate carboxykinase 2 [NM_213192]*	1,77	1,89	2,35
pcm1	*pericentriolar material 1 [NM_001115123]*	1,33	2,06	1,92

pcmtd2	*protein-L-isoaspartate (D-aspartate) O-methyltransferase domain containing 2 [NM_001039921]*	1,42	2,15	2,05
pdpk1a	*3-phosphoinositide dependent protein kinase-1a [NM_001077344]*	1,27	2,74	3,14
pdzrn4	*PDZ domain containing ring finger 4 [NM_001082923]*	1,07	3,03	2,62
peli2	*pellino homolog 2 (Drosophila) [NM_001002616]*	1,44	2,19	2,88
pias4b	*protein inhibitor of activated STAT, 4b [NM_200343]*	-1,08	3,54	2,22
plcb3	*phospholipase C, beta 3 (phosphatidylinositol-specific) [NM_001122773]*	2,05	3,02	4,18
pparaa	*peroxisome proliferator-activated receptor alpha a [NM_001161333]*	1,42	1,47	2,36
pparda	*peroxisome proliferator-activated receptor delta a*	1,43	2,69	3,25
pparab	*peroxisome proliferator activated receptor alpha b [NM_001102567]*	1,12	-1,88	-1,69
ppp6r2	*protein phosphatase 6, regulatory subunit 2 [NM_001025509]*	1,03	1,82	2,17
pppde1	*PPPDE peptidase domain containing 1 [NM_001003532]*	-1,13	1,82	1,61
prep	*prolyl endopeptidase [NM_001020753]*	-1,04	2,18	2,08
prkacbb	*protein kinase, cAMP-dependent, catalytic, beta b [NM_001034976]*	1,10	1,18	2,22
prkchb	*protein kinase C, eta, b [NM_001044806]*	1,00	1,65	4,48
ptk2.2	*protein tyrosine kinase 2b [NM_198819]*	1,34	1,08	2,57
ptprfb	*protein tyrosine phosphatase, receptor type, f, b [NM_001083576]*	2,21	2,15	2,68
rab12	*RAB12, member RAS oncogene family [NM_001020630]*	1,01	1,73	2,03
rabgef1l	*RAB guanine nucleotide exchange factor (GEF) 1, like [NM_200941]*	1,34	1,11	2,56
rassf2l	*Ras association (RalGDS/AF-6) domain family 2, like [NM_001002195]*	-1,09	1,96	1,77
rb1	*retinoblastoma 1 [NM_001077780]*	-1,12	3,00	1,93
rgmb	*RGM domain family, member B [NM_001001727]*	-1,06	1,41	2,02
rhogc	*ras homolog gene family, member Gc [NM_199692]*	1,18	2,15	1,53
ripply3	*ripply3 [BC165483]*	1,81	1,18	2,50
rnasekb	*ribonuclease, RNase K b [NM_001045375]*	1,47	2,98	1,54
robo2	*roundabout homolog 2 [NM_131633]*	2,54	1,57	2,43
rp2	*retinitis pigmentosa 2 (X-linked recessive) [NM_213446]*	1,12	2,29	1,49
rps26l	*ribosomal protein S26 like [CN170396]*	1,57	-1,35	2,29
rps6ka1	*ribosomal protein S6 kinase a, polypeptide 1 [NM_001077775]*	1,12	2,07	1,96
rxfp1	*relaxin/insulin-like family peptide receptor 1 [NM_001190934]*	1,69	-1,26	2,77

rxfp2	*relaxin/insulin-like family peptide receptor 2 [NM_200443]*	1,05	2,03	2,37
sb:cb113	*sb:cb113 [CT605248]*	1,63	-1,17	2,05
sb:cb120	*sb:cb120 [CN015641]*	1,77	1,06	-1,14
sb:cb288	*sb:cb288*	1,08	2,63	2,28
sb:cb429	*sb:cb429 [CT719690]*	-1,12	1,83	1,96
scg2a	*secretogranin II (chromogranin C) a [DY546328]*	1,41	-1,23	2,08
senp3b	*SUMO1/sentrin/SMT3 specific peptidase 3b [NM_001083842]*	-1,18	1,31	1,78
serpine2	*serine (or cysteine) proteinase inhibitor, clade E (nexin, plasminogen activator inhibitor type 1),member 2 [NM_200184]*	-1,07	2,59	2,01
setdb1b	*setdb1b [AW115964]*	1,15	1,07	2,08
sgsm3	*small G protein signaling modulator 3 [NM_213330]*	1,38	2,05	1,63
shc2	*SHC (Src homology 2 domain containing) transforming protein 2 [NM_001044973]*	1,67	2,85	2,36
si:ch1073-380l12.1	*hypothetical protein LOC553524 [BC092003]*	-1,61	2,33	1,90
si:ch211-158d24.4	*si:ch211-158d24.4 [NM_001123259]*	-1,24	3,00	1,96
si:ch211-3o3.7	*si:ch211-3o3.7 [CK694741]*	-1,75	2,04	4,09
si:ch73-52e5.2	*si:ch73-52e5.2 [NM_001122629]*	-1,38	1,83	1,91
si:dkey-218h11.4	*si:dkey-218h11.4 [NM_001044972]*	-1,30	1,66	1,33
si:dkey-21k4.4	*si:dkey-21k4.4 [NM_001099996]*	1,98	2,71	3,93
si:dkey-253d23.2	*zinc finger protein 347-like (LOC100007974) [XR_084440]*	1,02	1,88	2,50
si:dkey-267i17.5	*si:dkey-267i17.5 [NM_001111154]*	-1,11	1,33	2,64
si:dkeyp-117h8.1	*si:dkeyp-117h8.1 [NM_001199990]*	1,24	2,22	2,97
si:dkeyp-117h8.4	*si:dkeyp-117h8.4 (si:dkeyp-117h8.4), mRNA [NM_001082878]*	1,01	2,32	1,97
si:rp71-45k5.9	*si:rp71-45k5.9 [NM_001202440]*	1,66	2,37	4,18
siah2l	*seven in absentia homolog 2 (Drosophila)-like [NM_200427]*	1,18	1,57	2,08
six6a	*sine oculis-related homeobox 6a (six6a) [NM_201105]*	-1,33	1,79	2,89
slc16a1	*solute carrier family 16 (monocarboxylic acid transporters), member 1 [NM_200085]*	1,25	2,18	2,32
slc25a25a	*solute carrier family 25 (mitochondrial carrier; phosphate carrier), member 25a [NM_213257]*	1,59	2,80	3,02
slc25a25b	*solute carrier family 25 (mitochondrial carrier; phosphate carrier), member 25b [NM_001160020]*	1,71	3,52	3,21

slc25a3a	*solute carrier family 25 (mitochondrial carrier; phosphate carrier), member 3a [NM_200715]*	1,17	1,91	2,51
slc2a8	*solute carrier family 2 (facilitated glucose transporter), member 8 [NM_212798]*	-1,40	1,86	1,41
slc32a1	*solute carrier family 32 (GABA vesicular transporter), member 1 [NM_001080701]*	1,38	-1,26	2,63
slc34a2b	*solute carrier family 34 (sodium phosphate), member 2b [NM_182877]*	2,27	3,21	3,20
slc35b4	*solute carrier family 35, member B4 [NM_212652]*	1,24	2,23	3,65
slc44a4	*solute carrier family 44, member 4 [NM_200413]*	1,62	3,68	3,03
slc7a3	*solute carrier family 7 (cationic amino acid transporter, y+ system), member 3 [NM_001007329]*	2,72	6,14	3,28
smad9	*MAD homolog 9 (Drosophila)[NM_001004014]*	2,02	3,55	2,59
smarcal1	*SWI/SNF related, matrix associated, actin dependent regulator of chromatin, subfamily a-like 1 [NM_001127466]*	1,40	2,28	2,93
smtla	*somatolactin alpha [NM_001037706]*	2,04	1,31	4,18
smyhc1	*slow myosin heavy chain 1 [NM_001020507]*	1,84	-1,49	3,42
snx10a	*sorting nexin 10a [NM_001139462]*	-1,42	2,34	2,06
sort1a	*sortilin 1a [NM_213230]*	1,10	2,12	1,68
spice1	*spindle and centriole associated protein 1 [NM_001008636]*	-1,12	1,98	1,66
spint1b	*serine peptidase inhibitor, Kunitz type 1 b [NM_001111223]*	-1,13	3,02	1,86
src	*v-src sarcoma (Schmidt-Ruppin A-2) viral oncogene homolog (avian) [NM_001003837]*	1,20	2,53	1,99
ssx2ip	*synovial sarcoma, X breakpoint 2 interacting protein [NM_200835]*	-1,22	2,11	1,93
stk11ip	*serine/threonine kinase 11 interacting protein [NM_200288]*	1,32	1,75	2,47
stmn2a	*stathmin-like 2a [NM_001005923]*	1,72	1,03	3,77
stx3a	*syntaxin 3A [NM_200543]*	-1,19	2,30	2,16
sulf1	*sulfatase 1 [NM_001003846]*	1,15	2,38	2,47
svil	*supervillin [NM_001035261]*	-1,27	1,44	1,66
sybl1	*synaptobrevin-like 1 [NM_200266]*	-1,15	1,74	1,70
syt5a	*synaptotagmin Va [NM_001103137]*	2,67	6,11	11,07
tbce	*tubulin folding cofactor E [NM_001039989]*	-1,10	1,99	1,69
tbr1b	*T-box, brain, 1b[NM_001115090]*	-1,12	1,89	2,40
tdrd6	*Tudor domain-containing protein 6 [NM_001195281]*	-1,23	3,18	2,05

tectb	*tectorin beta [NM_001008594]*	-1,34	2,24	3,55
tfdp1b	*transcription factor Dp-1, b [NM_200776]*	1,26	2,04	1,90
tmem110l	*transmembrane protein 110, like [NM_200197]*	1,23	2,13	1,61
tmem150a	*transmembrane protein 150A [NM_001045410]*	2,00	1,16	2,55
tmem168a	*transmembrane protein 168a [NM_001083086]*	1,53	1,59	2,04
tmem175	*transmembrane protein 175 [NM_001100075]*	-1,13	3,24	1,59
tmem189	*transmembrane protein 189 [NM_001006052]*	1,09	2,13	1,91
tmem231	*transmembrane protein 231 [NM_200387]*	-1,22	1,92	1,73
tmem51b	*transmembrane protein 51b [NM_001102617]*	-1,18	1,12	2,06
tmprss4a	*transmembrane protease, serine 4a [NM_001077738]*	-1,53	2,14	2,44
tpte	*transmembrane phosphatase with tensin homology [NM_001025458]*	-1,01	2,24	1,81
trpm7	*transient receptor potential cation channel, subfamily M, member 7 [NM_001030061]*	1,58	1,82	2,46
tspan33	*tetraspanin 33 [NM_001002617]*	1,70	2,86	1,84
ube2b	*ubiquitin-conjugating enzyme E2B (RAD6 homolog) [NM_001002747]*	-1,08	1,70	2,04
ube2h	*ubiquitin-conjugating enzyme E2H (UBC8 homolog, yeast) [NM_201489]*	1,07	2,05	1,87
ube2j2	*ubiquitin-conjugating enzyme E2, J2 (UBC6 homolog, yeast) [NM_001089535]*	-1,00	1,97	2,07
uck2a	*uridine-cytidine kinase 2a [NM_001171067]*	-1,30	1,84	1,38
ucp2	*uncoupling protein 2 [NM_131176]*	1,12	3,50	2,11
ugt5a1	*UDP glucuronosyltransferase 5 family, polypeptide A1 [NM_001037420]*	4,68	7,73	11,04
ust	*uronyl-2-sulfotransferase [NM_001032729]*	1,61	3,82	2,78
uts2a	*urotensin 2, alpha [NM_212848]*	1,83	1,54	2,93
vps33a	*vacuolar protein sorting 33A [NM_001099973]*	1,51	2,38	2,12
waca	*WW domain containing adaptor with coiled-coil a [NM_199660]*	1,76	1,12	2,75
wu:fb51f10	*wu:fb51f10 [CN319073]*	2,60	1,27	1,56
wu:fb73e03	*wu:fb73e03 [AI558545]*	-1,08	1,38	2,15
wu:fb75h10	*wu:fb75h10 [BC090445]*	1,36	1,03	2,11
wu:fc41f02	*wu:fc41f02 [AI667541]*	1,64	-1,95	3,34
wu:fd07d03	*wu:fd07d03 [AI957871]*	2,12	1,20	3,51
wu:fd13g05	*wu:fd13g05 [EE713622]*	-1,20	1,30	1,82
wu:fj29e05	*wu:fj29e05 [AW232739]*	1,58	2,94	5,26

wu:fj53a09	*wu:fj53a09 [AW281608]*	1,60	-1,05	3,27
wu:fj58g06	*wu:fj58g06 [BC122233]*	1,07	2,16	1,62
wu:fj80e11	*wu:fj80e11 [AW420361]*	-1,09	-1,37	6,30
xrcc1	*X-ray repair complementing defective repair in Chinese hamster cells 1 [NM_001003988]*	-1,06	2,16	1,90
xrcc3	*X-ray repair complementing defective repair in Chinese hamster cells 3 [NM_001013541]*	1,14	2,01	1,28
yipf1	*Yip1 domain family, member 1 [NM_001003991]*	1,41	2,31	1,57
zfand5a	*zinc finger, AN1-type domain 5a[NM_213039]*	1,36	2,01	1,64
zgc:100937	*zgc:100937 [NM_001002744]*	1,03	2,17	1,59
zgc:101100	*zgc:101100 [NM_001003563]*	1,68	1,74	2,34
zgc:101606	*zgc:101606 [NM_001007457]*	1,53	2,65	3,53
zgc:101848	*zgc:101848 [NM_001005966]*	2,04	1,15	3,27
zgc:103558	*zgc:103558 [NM_001008600]*	-1,53	2,95	2,37
zgc:103670	*zgc:103670 [NM_001004664]*	1,13	1,55	2,05
zgc:110343	*zgc:110343 [NM_001013471]*	1,16	2,46	1,98
zgc:110667	*zgc:110667 [NM_001018146]*	-1,45	2,82	1,83
zgc:110779	*zgc:110779 [NM_001013318]*	-1,24	1,80	1,86
zgc:112023	*zgc:112023 [NM_001017673]*	1,59	2,56	2,96
zgc:112097	*zgc:112097 [NM_001159821]*	-1,40	1,81	1,36
zgc:113071	*zgc:113071 [NM_001014333]*	-1,20	2,05	1,70
zgc:113223	*zgc:113223 [NM_001013551]*	2,12	2,63	-1,17
zgc:113516	*zgc:113516 [NM_001013574]*	1,27	3,34	3,14
zgc:114199	*zgc:114199 [NM_001030149]*	1,07	2,52	1,55
zgc:123246	*zgc:123246 [NM_001037694]*	1,14	2,93	2,58
zgc:123285	*zgc:123285 [BC109444]*	-1,59	1,47	1,83
zgc:152869	*zgc:152869 [NM_001077167]*	-1,12	1,81	1,31
zgc:152968	*zgc:152968 [NM_001077565]*	1,04	2,14	1,81
zgc:153102	*zgc:153102 [NM_001077310]*	2,26	6,28	6,20
zgc:153125	*zgc:153125 [NM_001077745]*	1,14	1,71	2,01
zgc:153329	*zgc:153329 [NM_001077179]*	1,31	1,82	2,43
zgc:153383	*zgc:153383 [NM_001045461]*	-1,10	1,97	2,14

zgc:153436	*zgc:153436 [NM_001044847]*	-1,23	1,55	1,88
zgc:153463	*zgc:153463 [NM_001076644]*	-1,17	2,21	1,94
zgc:153674	*zgc:153674 [NM_001045356]*	-1,61	3,42	2,05
zgc:153721	*zgc:153721 [NM_001079680]*	1,23	1,28	2,72
zgc:153788	*zgc:153788 [NM_001045347]*	1,17	2,62	2,81
zgc:153911	*zgc:153911 [NM_001077315]*	1,32	2,46	1,91
zgc:153955	*zgc:153955 [NM_001080001]*	2,04	2,32	2,77
zgc:153990	*zgc:153990 [NM_001077271]*	-1,08	2,01	1,48
zgc:154086	*zgc:154086 [NM_001077327]*	-1,21	2,15	1,59
zgc:154116	*zgc:154116 [NM_001077259]*	-1,21	1,75	1,97
zgc:158219	*zgc:158219 [NM_001081574]*	1,46	3,16	1,54
zgc:158267	*zgc:158267 [NM_001080072]*	1,37	1,08	2,37
zgc:158281	*zgc:158281 [NM_001089403]*	1,35	2,47	1,69
zgc:158328	*zgc:158328 [NM_001080189]*	-1,02	-1,14	1,85
zgc:158432	*zgc:158432 [NM_001079957]*	1,07	1,42	2,05
zgc:158624	*zgc:158624 [NM_001080672]*	1,74	2,60	2,80
zgc:158642	*zgc:158642 [NM_001080624]*	-1,39	1,92	2,12
zgc:158742	*zgc:158742 [NM_001081691]*	1,38	1,77	2,13
zgc:158823	*zgc:158823 [NM_001089556]*	-1,10	2,91	5,31
zgc:162229	*zgc:162229 [BC091899]*	1,22	-1,15	2,71
zgc:162271	*zgc:162271 [NM_001089373]*	1,11	2,05	2,06
zgc:162939	*zgc:162939 [NM_001123258]*	1,04	2,12	2,25
zgc:162952	*zgc:162952 [NM_001089574]*	1,07	3,65	2,26
zgc:162958	*zgc:162958 [NM_001089338]*	-1,11	2,25	1,82
zgc:165555	*zgc:165555 [NM_001098761]*	1,63	2,05	2,05
zgc:171516	*zgc:171516 [NM_001105691]*	1,54	2,79	2,48
zgc:171813	*zgc:171813 [NM_001113611]*	1,04	2,57	2,53
zgc:172101	*zgc:172101 [NM_001113644]*	1,40	2,16	2,82
zgc:172206	*zgc:172206 [NM_001110400]*	1,09	4,18	6,24
zgc:172271	*zgc:172271 [NM_001114466]*	-1,02	2,00	1,73

zgc:172282	*zgc:172282 [NM_001113649]*	1,42	1,05	4,60
zgc:173594	*zgc:173594 [NM_001109854]*	1,42	2,80	2,35
zgc:175133	*zgc:175133 [NM_001127471]*	1,12	2,65	2,52
zgc:175284	*zgc:175284 [NM_001113614]*	1,66	-1,02	2,50
zgc:194125	*zgc:194125 [NM_001130667]*	1,51	2,69	2,30
zgc:194851	*zgc:194851 [NM_001128698]*	1,41	2,31	2,32
zgc:195212	*zgc:195212 [NM_001135145]*	-1,11	1,25	1,83
zgc:198419	*zgc:198419 [NM_001113659]*	1,22	3,36	2,46
zgc:55292	*zgc:55292 [NM_199546]*	-1,05	1,91	1,90
zgc:56429	*zgc:56429 [NM_200277]*	-1,19	1,68	2,00
zgc:64119	*zgc:64119 [NM_200379]*	1,25	1,20	2,03
zgc:64213	*zgc:64213 [NM_200421]*	1,57	1,28	2,60
zgc:65781	*zgc:65781 [NM_199632]*	1,26	-1,21	2,32
zgc:66299	*zgc:66299 [NM_200953]*	1,42	1,67	2,29
zgc:66430	*zgc:66430 [NM_200055]*	-1,16	2,00	1,93
zgc:77147	*zgc:77147 [NM_205646]*	1,95	1,48	3,40
zgc:77375	*zgc:77375 [NM_205631]*	1,31	3,06	4,49
zgc:77828	*zgc:77828 [NM_212896]*	1,29	2,30	2,38
zgc:85964	*zgc:85964 [NM_001005928]*	1,40	2,82	3,68
zgc:86759	*zgc:86759 [NM_001002132]*	-2,02	2,03	3,06
zgc:86764	*zgc:86764[NM_001002684]*	1,31	1,02	4,93
zgc:91890	*zgc:91890 [NM_001002182]*	1,14	1,47	-1,45
zgc:92658	*zgc:92658 [NM_001002426]*	3,71	1,05	4,74
zgc:92689	*zgc:92689 [NM_001002409]*	1,41	2,01	1,92
zgc:92723	*zgc:92723 [NM_001002583]*	1,78	5,53	2,64
znf296	*zinc finger protein 296 [NM_001044319]*	-1,17	1,57	1,97
znfl2a	*zinc finger-like gene 2a [NM_194390]*	1,65	1,08	2,20
zranb1b	*zinc finger, RAN-binding domain containing 1b [NM_001077768]*	1,31	1,98	2,09

Common to packing and transport

Gene symbol	Gene name	Fold change*		
		**0 h**	**48 h**	**72 h**
abhd4	*abhydrolase domain containing 4 [NM_001017613]*	2,02	2,05	1,74
agxtb	*alanine-glyoxylate aminotransferase b [NM_213162]*	2,03	1,60	1,47
alp	*alkaline phosphatase [NM_201007]*	1,95	2,09	1,97
apobl	*apolipoprotein B, like [BC090504]*	1,77	1,25	2,18
asb1	*ankyrin repeat and SOCS box-containing 1[NM_001002736]*	2,05	1,35	1,94
atxn1a	*ataxin 1a [NM_001044826]*	2,30	2,96	5,45
bcl2l11	*BCL2-like 11 [NM_001135791]*	2,13	2,30	2,68
ca5	*carbonic anhydrase V [NM_001111201]*	2,13	2,47	2,05
capn5a	*calpain 5a [NM_001080007]*	1,86	1,25	3,16
caspc	*caspase c [NM_001013502]*	3,18	2,54	3,32
cda	*cytidine deaminase [NM_205679]*	2,62	1,94	2,11
cdc14ab	*CDC14 cell division cycle 14 homolog A, b [NM_001128538]*	2,15	1,57	2,44
cdkn1d	*cyclin-dependent kinase inhibitor 1D [BC124236]*	3,14	4,24	3,78
col4a3bp	*collagen, type IV, alpha 3 (Goodpasture antigen) binding protein [NM_001017914]*	1,64	2,57	2,23
cry2b	*cryptochrome 2b [NM_131792]*	3,91	2,51	2,28
cyp3a65	*cytochrome P450, family 3, subfamily A, polypeptide 65 [NM_001037438]*	3,08	4,62	4,61
dcaf6	*ddb1 and cul4 associated factor 6 [NM_001145090]*	1,95	2,17	2,54
dtng	*dystrobrevin, gamma [NM_199709]*	1,96	2,00	2,56
egfra	*epidermal growth factor receptor a (erythroblastic leukemia viral (v-erb-b) oncogene homolog, avian) [NM_194424]*	2,33	2,68	4,20
elovl1b	*elongation of very long chain fatty acids (FEN1/Elo2, SUR4/Elo3, yeast)-like 1b [NM_213416]*	1,91	2,23	1,44

eng1b	*engrailed 1b [NM_001013498]*	2,48	2,57	2,30
epha2	*eph receptor A2 [NM_131415]*	2,21	1,98	1,56
fam20b	*family with sequence similarity 20, member B (H. sapiens) [NM_001044818]*	1,80	2,53	2,25
fbp2	*fructose-1,6-bisphosphatase 2 (fbp2) [NM_001004008]*	1,95	1,12	2,25
fem1c	*fem-1 homolog c (C.elegans) [NM_198145]*	3,45	2,96	3,04
fhla	*four and a half LIM domains a (fhla) [NM_001007287]*	4,10	2,58	1,78
fn1b	*fibronectin 1b [NM_001013261]*	2,80	1,61	2,13
fos	*v-fos FBJ murine osteosarcoma viral oncogene homolog [NM_205569]*	6,52	4,07	1,35
foxp1a	*forkhead box P1a [NM_001077564]*	2,63	2,44	1,78
gadd45ba	*growth arrest and DNA-damage-inducible, beta a [NM_213031]*	5,46	6,75	4,68
gadd45g	*growth arrest and DNA-damage-inducible, gamma [NM_205691]*	4,71	9,64	6,58
gbgt1l3	*globoside alpha-1,3-N-acetylgalactosaminyltransferase 1, like 3 [NM_001004620]*	3,04	1,69	2,48
gnav1	*guanine nucleotide binding protein (G protein) alpha v1 [NM_001166014]*	1,82	2,08	1,78
igfbp1a	*insulin-like growth factor binding protein 1a [NM_173283]*	7,64	8,66	6,48
junbl	*jun B proto-oncogene, like [NM_212750]*	5,37	8,37	3,65
keap1b	*kelch-like ECH-associated protein 1b [NM_001113477]*	1,99	1,38	2,90
LOC100002910	*novel protein containing lectin C-type domains [NM_001100081]*	3,47	1,91	3,32
LOC100151215	*phosphodiesterase 7A-like (LOC100151215) [NM_001168263]*	2,62	2,17	2,98
LOC568915	*novel protein similar to H.sapiens GALNTL6 [NM_001145602]*	1,94	1,92	2,65
mknk2b	*MAP kinase-interacting serine/threonine kinase 2b [NM_194402]*	2,98	2,26	3,10
mll	*myeloid/lymphoid or mixed-lineage leukemia (trithorax homolog, Drosophila) [NM_001110279]*	1,80	1,72	2,40
msrb2	*methionine sulfoxide reductase B2 [NM_212921]*	1,41	1,69	2,09
ncbp2	*nuclear cap binding protein subunit 2 [EH576001]*	2,81	1,42	3,28
negr1	*neuronal growth regulator 1 [NM_001003851]*	2,27	2,80	2,67
nfe2l1	*nuclear factor, erythroid derived 2,-like 1 [NM_212855]*	1,74	3,26	2,42
nfyc	*nuclear transcription factor Y, gamma [NM_199639]*	1,39	2,11	2,28
notch1b	*notch homolog 1b [NM_131302]*	2,06	1,41	3,06
nr1h5	*nuclear receptor subfamily 1, group H, member 5 [NM_001123241]*	3,04	1,15	2,08

nrxn1b	*neurexin 1b [NM_001079959]*	2,67	3,00	4,03
nt5c2l1	*5'-nucleotidase, cytosolic II, like 1 [NM_001004549]*	2,03	2,36	2,26
onecutl	*one cut domain, family member, like [NM_152984]*	3,06	3,01	3,84
pnp6	*purine nucleoside phosphorylase 6 [NM_205655]*	7,35	10,00	16,29
pnrc2	*proline-rich nuclear receptor coactivator 2 [NM_001130192]*	2,04	1,89	1,91
pptc7	*PTC7 protein phosphatase homolog (S. cerevisiae) [NM_001007378]*	3,22	2,15	3,17
ptk2.1	*protein tyrosine kinase 2a [NM_131796]*	2,16	1,30	2,92
ptprfb	*protein tyrosine phosphatase, receptor type, f, b [NM_001083576]*	2,21	2,15	2,68
robo2	*roundabout homolog 2 [NM_131633]*	2,54	1,57	2,43
scg3	*secretogranin III [NM_200757]*	2,35	2,65	1,24
sec14l1	*SEC14-like 1 (S. cerevisiae) [NM_201098]*	2,69	1,74	1,79
sema6dl	*sema domain, transmembrane domain (TM), and cytoplasmic domain, (semaphorin) 6D, like [NM_001017682]*	2,42	2,80	3,63
sgk2a	*serum/glucocorticoid regulated kinase 2a [NM_001113622]*	1,46	1,77	2,48
si:ch211-219a4.3	*si:ch211-219a4.3 [NM_001114447]*	2,38	-1,02	3,20
si:ch211-225b11.1	*si:ch211-225b11.1[NM_001110461]*	1,84	2,16	2,80
si:dkey-12h9.13	*si:dkey-12h9.13, mRNA [BC134093]*	2,07	1,57	2,79
si:dkey-205h13.1	*si:dkey-205h13.1 [NM_001195247]*	2,18	2,07	2,34
si:dkey-46l15.1	*si:dkey-46l15.1 [NM_001123252]*	1,57	2,03	1,79
si:dkey-52h23.1	*si:dkey-52h23.1[NM_001197058]*	5,50	7,34	4,40
si:dkey-5g7.3	*si:dkey-5g7.3 [NM_001025529]*	1,93	2,67	3,31
slc1a2b	*solute carrier family 1 (glial high affinity glutamate transporter), member 2b [NM_199979]*	1,52	-1,23	2,18
slc26a5	*solute carrier family 26, member 5 [NM_201473]*	2,80	4,52	3,87
slc27a6	*solute carrier family 27 (fatty acid transporter), member 6 [NM_001077386]*	4,21	7,69	11,30
slc34a2b	*solute carrier family 34 (sodium phosphate), member 2b [NM_182877]*	2,27	3,21	3,20
slit2	*slit (Drosophila) homolog 2 [NM_131735]*	2,67	4,55	2,80
smg7	*Smg-7 homolog, nonsense mediated mRNA decay factor (C. elegans) [NM_001045083]*	1,86	1,18	2,33
spoplb	*speckle-type POZ protein-like b [NM_001079969]*	1,70	1,96	2,83
sycp1	*synaptonemal complex protein 1 mRNA [NM_001118894]*	10,81	11,54	12,34

synm	*synemin, intermediate filament protein [NM_001044875]*	3,22	3,54	4,73
tcf7	*transcription factor 7 (T-cell specific, HMG-box) [NM_001012389]*	2,32	1,12	1,95
tdh	*L-threonine dehydrogenase (tdh) [NM_213245]*	2,70	2,09	1,75
tesk2	*testis-specific kinase 2 [NM_001110476]*	2,36	2,49	2,49
tgm2b	*transglutaminase 2b (tgm2b) [NM_212656]*	1,75	2,09	1,37
tmem86a	*transmembrane protein 86A [NM_001013512]*	3,24	1,95	3,42
tpd52l1	*tumor protein D52-like 1 (tpd52l1)[NM_001020734]*	2,89	2,40	1,85
ucp1	*uncoupling protein 1[NM_199523]*	2,18	2,45	2,10
vim	*vimentin [NM_131872]*	2,27	1,94	2,62
wnt5b	*wingless-type MMTV integration site family, member 5b [NM_130937]*	5,24	5,75	4,99
wu:fj37h01	*wu:fj37h01 [EB941263]*	2,15	2,32	1,33
wu:fj62d01	*wu:fj62d01 [EH506070]*	1,88	2,25	1,30
wu:fk57a03	*wu:fk57a03 [EH995454]*	2,48	1,52	2,14
ypel3	*yippee-like 3 [NM_212790]*	2,03	1,22	1,22
zcchc8	*zinc finger, CCHC domain containing 8 [NM_001083818]*	1,75	1,99	2,71
zer1	*zer-1 homolog (C. elegans) (zer1)[NM_212901]*	2,14	1,94	1,54
zgc:103657	*zgc:103657 [NM_001005948]*	4,73	1,69	5,38
zgc:110326	*zgc:110326 [NM_001017805]*	1,86	-1,08	2,14
zgc:112101	*zgc:112101 [NM_001017748]*	2,87	3,66	3,84
zgc:136474	*zgc:136474 [NM_001045308]*	2,94	3,47	3,92
zgc:153047	*zgc:153047 [NM_001076720]*	2,22	2,13	2,38
zgc:154055	*zgc:154055 [NM_001077142]*	2,46	1,81	2,57
zgc:158263	*zgc:158263 [NM_001080679]*	2,24	2,92	2,50
zgc:158299	*zgc:158299 [NM_001080082]*	1,95	2,02	3,18
zgc:162126	*zgc:162126 [NM_001082921]*	3,73	4,31	2,66
zgc:162129	*zgc:162129 [NM_001083570]*	2,16	1,87	1,83
zgc:162584	*zgc:162584 [NM_001098189]*	1,96	1,37	2,42
zgc:162730	*zgc:162730 [NM_001089404]*	3,83	2,42	1,63
zgc:172238	*zgc:172238 [NM_001113635]*	1,24	-1,87	-1,42
zgc:198195	*zgc:198195 [NM_001079704]*	1,84	1,70	2,13

zgc:63907	*zgc:63907 [NM_201137]*	1,72	2,28	2,07
zgc:63938	*zgc:63938 [NM_200346]*	2,53	1,58	1,75
zgc:65870	*zgc:65870[NM_213117]*	2,15	4,90	4,59
zgc:66488	*zgc:66488 [NM_200431]*	1,78	1,38	2,55
zgc:73324	*zgc:73324[NM_200789]*	3,80	5,06	3,04
zgc:92066	*zgc:92066 [NM_001002378]*	1,56	2,62	2,11
zgc:92090	*zgc:92090 [NM_001002367]*	2,98	4,33	3,92
zgc:92658	*zgc:92658 [NM_001002426]*	3,71	1,05	4,74

List of genes that are significantly upregulated immediately after packing (0 h), and at 48 and 72 h during transport compared to the expression in liver of fish prior to transport (basal or pre-stressed). * Fold-changes (positive for upregulated and negative for downregulated genes) are calculated as the ratio of the gene expression level at a particular time point to the respective basal levels.

Additional file 4: Table S4 Full list of genes that are significantly downregulated in the zebrafish liver during transport process.

Only after packing (0 h)

**Gene symbol**	**Gene name**	**Fold change***		
		**0 h**	**48 h**	**72 h**
abcg1	*ATP-binding cassette, sub-family G (WHITE), member 1 [NM_001110454]*	-2,75	1,01	1,03
bmpr1ba	*bone morphogenetic protein receptor, type 1ba [NM_131457]*	-2,41	1,82	1,04
btaf1	*BTAF1 RNA polymerase II, B-TFIID transcription factor-associated [NM_001159406]*	-1,95	-1,11	1,44
dck	*deoxycytidine kinase [NM_001007056]*	-2,18	1,45	-1,62
dnajc21	*DnaJ (Hsp40) homolog, subfamily C, member 21 [NM_200044]*	-1,87	1,14	1,19
igiv1s8	*immunoglobulin light iota variable 1, s8 [AF246189]*	-3,39	1,10	1,35
LOC100007939	*hypothetical LOC100007939 [XM_001346237]*	-3,17	-1,13	-1,46
morn4	*MORN repeat containing 4 [NM_001017559]*	-2,72	1,29	1,37
nxph1	*neurexophilin 1 [NM_001002733]*	-3,45	1,81	1,92
pacs1	*phosphofurin acidic cluster sorting protein 1 [NM_001098748]*	-1,41	1,91	1,66
pcgf6	*polycomb group ring finger 6 [NM_001089369]*	-1,64	1,24	-1,05
prdm14	*PR domain containing 14 [NM_001163831]*	-2,61	-1,71	1,76
pygo2	*pygopus homolog 2 (Drosophila) [NM_001033111]*	-1,90	1,35	1,08
si:busm1-163l24.4	*si:busm1-163l24.4 [NM_001005315]*	-8,30	1,40	2,09
si:ch211-63o20.7	*si:ch211-63o20.7 [NM_001045121]*	-2,57	-1,16	1,09
si:dkey-7n6.2	*si:dkey-7n6.2 [NM_001199998]*	-4,26	1,35	1,48
slc17a5	*solute carrier family 17 (anion/sugar transporter), member 5 [NM_001076727]*	-2,47	1,31	-1,12
slc17a9b	*solute carrier family 17, member 9b [NM_001002635]*	-1,60	1,25	1,01
sp8b	*sp8 transcription factor b[NM_205550]*	-4,23	1,71	2,31
stx11b.2	*syntaxin 11b.2 [NM_001105602]*	-3,08	-1,15	2,23
zgc:100947	*zgc:100947 [NM_001003624]*	-4,61	1,29	1,11
zgc:113143	*zgc:113143 [NM_001012500]*	-2,17	1,34	1,36

zgc:153115	*zgc:153115 [NM_001077329]*	-1,86	1,05	1,17
zgc:153381	*zgc:153381 [NM_001077605]*	-2,64	1,19	1,36
zgc:66443	*zgc:66443 [NM_213535]*	-2,39	4,54	1,32
zgc:73359	*zgc:73359 [NM_200837]*	-2,18	1,08	1,57

Only during transport (at 48 and/or 72 h)

**Gene symbol**	**Gene name**	**Fold change***		
		**0 h**	**48 h**	**72 h**
acadl	*acyl-Coenzyme A dehydrogenase, long chain [NM_201181]*	1,31	-1,38	-1,60
acadm	*acyl-Coenzyme A dehydrogenase, C-4 to C-12 straight chain [NM_213089]*	1,43	-1,29	-1,45
acads	*acyl-Coenzyme A dehydrogenase, C-2 to C-3 short chain [NM_001003743]*	1,47	-1,65	-1,46
acox1	*acyl-Coenzyme A oxidase 1, palmitoyl [NM_001005933]*	1,37	-1,69	-1,40
acy3.2	*aspartoacylase (aminocyclase) 3.2 [NM_001002347]*	1,44	-1,89	-1,60
add1	*adducin 1 (alpha) [NM_001079958]*	1,15	-1,91	-1,74
adh5	*alcohol dehydrogenase 5[NM_131849]*	1,22	-1,55	-1,74
adh8a	*alcohol dehydrogenase 8a [NM_001001946]*	1,57	-2,41	-5,77
adh8b	*alcohol dehydrogenase 8b [NM_203460]*	1,48	-2,74	-2,68
agl	*amylo-1, 6-glucosidase, 4-alpha-glucanotransferase [NM_001172653]*	1,50	-1,92	-1,42
agmat	*agmatine ureohydrolase (agmatinase) [NM_001077551]*	-1,13	-3,87	-2,67
agxt2l1	*alanine-glyoxylate aminotransferase 2-like 1 [NM_200449]*	1,19	-2,24	-2,17
ak4	*adenylate kinase 4 [NM_213299]*	1,17	-2,26	-1,80
akap1b	*A kinase (PRKA) anchor protein 1b [NM_001098179]*	1,11	-1,81	-1,31
akr1a1a	*aldo-keto reductase family 1, member A1a (aldehyde reductase) [NM_001003783]*	1,02	-2,47	-1,90
alas1	*aminolevulinate, delta-, synthetase 1 [NM_201287]*	-1,13	-2,74	-2,07
alas2	*aminolevulinate, delta-, synthetase 2 [NM_131682]*	1,26	-2,37	-1,83
aldh16a1	*aldehyde dehydrogenase 16 family, member A1 [NM_001079954]*	1,46	-2,97	-1,09
aldh2b	*aldehyde dehydrogenase 2b [NM_213301]*	1,54	-1,68	-1,80
aldh4a1	*aldehyde dehydrogenase 4 family, member A1 [NM_201158]*	1,11	-1,97	-1,54

aldh6a1	*aldehyde dehydrogenase 6 family, member A1 [NM_001002374]*	1,32	-1,42	-1,52
aldh7a1	*aldehyde dehydrogenase 7 family, member A1 [NM_212724]*	1,44	-1,60	-1,80
aldh9a1a	*aldehyde dehydrogenase 9 family, member A1a [NM_201471]*	1,43	-2,15	-2,95
amd1	*adenosylmethionine decarboxylase 1 [NM_001161601]*	1,16	-1,46	-1,90
amt	*aminomethyltransferase [NM_001006021]*	-1,34	-2,27	-1,59
amy2a	*amylase, alpha 2A; pancreatic [NM_213011]*	1,42	-1,36	-3,21
angptl3	*angiopoietin-like 3 [NM_131818]*	-1,19	-4,05	-4,30
ank1	*ankyrin 1, erythrocytic [NM_001005969]*	1,39	-5,24	-4,62
ap1b1	*adaptor-related protein complex 1, beta 1 subunit [NM_001128530]*	-1,30	-2,20	-2,17
ap4s1	*adaptor-related protein complex 4, sigma 1 subunit [NM_001003826]*	-1,04	-2,36	-2,44
aplnrb	*Danio rerio apelin receptor b [NM_001030197]*	1,13	-1,86	-1,59
aprt	*adenine phosphoribosyl transferase [NM_200668]*	-1,09	-2,02	-2,08
aqp10a	*aquaporin 10a [NM_001002349]*	1,81	-1,81	-1,25
aqp12	*aquaporin 12 [BC095564]*	-1,07	-2,19	-2,41
aqp8a.1	*aquaporin 8a, tandem duplicate 1 [NM_001004661]*	-1,09	-3,83	-4,01
ar	*androgen receptor [NM_001083123]*	-1,18	-2,73	-2,26
araf	*v-raf murine sarcoma 3611 viral oncogene homolog [NM_205743]*	1,13	-1,99	-1,66
asah1b	*N-acylsphingosine amidohydrolase (acid ceramidase) 1b [NM_200577]*	1,58	-1,73	-1,77
asns	*asparagine synthetase [NM_201163]*	-1,53	-2,20	-2,66
atp11c	*ATPase, Class VI, type 11C [DT880386]*	1,19	-2,30	-1,74
auh	*AU RNA binding protein/enoyl-Coenzyme A hydratase [NM_001003576]*	1,05	-2,04	-1,80
ba1	*ba1 globin [NM_131020]*	1,37	-1,61	-1,61
ba1l	*ba1 globin, like [NM_001013027]*	1,29	-1,63	-1,65
bccip	*BRCA2 and CDKN1A interacting protein [NM_001013475]*	-1,01	-2,01	-2,18
bckdk	*branched chain alpha-ketoacid dehydrogenase kinase [NM_213060]*	-1,39	-2,40	-2,33
bdh2	*3-hydroxybutyrate dehydrogenase, type 2 [NM_001017809]*	-1,23	-2,43	-2,37
bhlha15	*basic helix-loop-helix family, member a15 [NM_001077652]*	-1,48	-2,69	-3,14
bhlhe40	*basic helix-loop-helix family, member e40 [NM_212679]*	1,49	-1,78	-1,58
brd2a	*bromodomain-containing 2a [NM_131200]*	-1,18	-1,57	-2,10
btbd6a	*BTB (POZ) domain containing 6a[NM_001114925]*	1,14	-2,00	1,31

c1qtnf1	*C1q and tumor necrosis factor related protein 1 [NM_001017875]*	1,06	-2,30	-2,62
c1qtnf5	*C1q and tumor necrosis factor related protein 5 [NM_001029953]*	1,07	-1,67	-1,94
c3b	*complement component C3B [AF047414]*	1,45	-3,59	-3,33
c3c	*complement component c3c [NM_001037236]*	1,25	-2,54	-2,21
c6ast3	*six-cysteine containing astacin protease 3 [NM_001013526]*	1,54	-1,62	-3,49
c6ast4	*six-cysteine containing astacin protease 4 [NM_001025180]*	-1,01	-2,37	-5,64
cahz	*carbonic anhydrase [NM_131110]*	1,35	-2,71	-2,31
cav1	*caveolin 1 [BC115201]*	1,05	-2,55	-2,75
cbln4	*cerebellin 4 precursor [NM_001110391]*	1,97	-2,58	-1,26
cbr1	*carbonyl reductase 1 [NM_194406]*	1,40	-1,82	-1,14
cbsa	*cystathionine-beta-synthase a [NM_001111232]*	1,13	-1,82	-1,42
cbx1b	*chromobox homolog 1b (HP1 beta homolog Drosophila) [NM_001002090]*	1,05	-1,93	-1,69
ccnd1	*cyclin D1 [NM_131025]*	-1,53	-2,33	-2,14
ccpg1	*cell cycle progression 1 [NM_001007426]*	-1,09	-2,37	-1,93
cdkn3	*cyclin-dependent kinase inhibitor 3 [NM_001145681]*	-1,68	-1,90	-2,01
cdkrap3	*CDK5 regulatory subunit associated protein 3 [NM_001002105]*	-1,41	-1,51	-2,14
cdo1	*cysteine dioxygenase, type I [NM_200741]*	1,50	-3,33	-2,90
cebpa	*CCAAT/enhancer binding protein (C/EBP), alpha [NM_131885]*	-1,99	-2,80	-3,04
cel.1	*carboxyl ester lipase, tandem duplicate 1 [NM_199607]*	1,38	-1,29	-2,44
cel.2	*carboxyl ester lipase (danio-celip) [AF003943]*	2,17	1,10	-2,00
ces2	*carboxylesterase 2 (intestine, liver) [NM_001077252]*	1,78	-1,89	-1,77
ces3	*carboxylesterase 3 [NM_001044936]*	1,52	-1,82	-1,61
chka	*choline kinase alpha [NM_001044865]*	-1,41	-3,50	-3,23
cilp	*cartilage intermediate layer protein, nucleotide pyrophosphohydrolase [NM_001199362]*	-1,04	-3,16	-2,85
cish	*cytokine inducible SH2-containing protein [NM_001076617]*	1,31	-1,89	-1,27
cldn2	*claudin 2 [BC081634]*	1,20	-1,61	-1,82
clptm1l	*cleft lip and palate associated transmembrane protein 1, like [NM_001002380]*	1,05	-1,86	-2,20
col1a1a	*collagen, type I, alpha 1a [NM_199214]*	1,14	-2,31	-4,10
col1a1b	*collagen, type I, alpha 1b [NM_201478]*	1,25	-2,08	-4,31

col1a2	*collagen, type I, alpha 2 [NM_182968]*	-1,07	-3,53	-7,14
col5a2l	*collagen, type V, alpha 2-like [NM_001145782]*	-1,12	-2,28	-2,63
colec11	*collectin sub-family member 11 [NM_001007331]*	1,48	-1,99	-1,53
comta	*catechol-O-methyltransferase a[NM_001030157]*	1,08	-1,97	-1,58
cpt2	*carnitine palmitoyltransferase II [NM_001007447]*	1,01	-2,23	-1,70
crbn	*cereblon. [BC080253]*	1,67	-1,46	-1,74
cry1a	*cryptochrome 1a [NM_001077297]*	2,17	-2,38	-2,89
cryaa	*crystallin, alpha A [NM_152950]*	1,18	-4,21	-4,50
crym	*crystallin, mu [NM_001080687]*	1,12	-2,69	-2,40
cthl	*cystathionase (cystathionine gamma-lyase), like[NM_001005400]*	1,33	-2,39	-1,47
cutc	*cutC copper transporter homolog (E. coli) [NM_001006046]*	-1,09	-2,20	-2,11
cx30.3	*connexin 30.3 [NM_212825]*	1,34	1,01	-2,05
cyb5a	*cytochrome b5 type A (microsomal) [NM_213135]*	1,45	-1,56	-1,45
cyb5r3	*cytochrome b5 reductase 3 [NM_212685]*	1,20	-1,79	-1,38
cyp1a	*cytochrome P450, family 1, subfamily A [NM_131879]*	1,08	-2,28	-1,80
cyp1c1	*cytochrome P450, family 1, subfamily C, polypeptide 1 [NM_001020610]*	1,09	-2,87	-3,16
cyp26b1	*cytochrome P450, family 26, subfamily b, polypeptide 1 [NM_212666]*	-1,29	-2,29	-3,04
cyp2ad2	*cytochrome P450, family 2, subfamily AD, polypeptide 2 [NM_152954]*	1,30	-3,02	-1,82
cyp2u1	*cytochrome P450, family 2, subfamily U, polypeptide 1 [NM_001145564]*	1,18	-2,32	-2,17
cyp2x10.2	*cytochrome P450, family 2, subfamily X, polypeptide 10.2 [NM_001044309]*	1,64	-1,60	-1,27
cyp39a1	*cytochrome P450, family 39, subfamily A, polypeptide 1 [NM_001030189]*	1,42	-2,84	-4,94
cyp3c4	*cytochrome P450, family 3, subfamily C, polypeptide 4 [NM_001077548]*	1,45	-3,01	-1,95
cyp4v2	*cytochrome P450, family 4, subfamily V, polypeptide 2 [NM_001077602]*	1,40	-1,81	-1,42
cyp7a1a	*cytochrome P450, family 7, subfamily A, polypeptide 1a [NM_201173]*	1,48	-6,93	-10,02
cyr61	*cysteine-rich, angiogenic inducer, 61 [NM_001080987]*	-1,02	-1,76	-2,26
dao.1	*D-amino-acid oxidase 1 [NM_001033740]*	1,09	-2,88	-3,30
dao.2	*D-amino-acid oxidase 2 [NM_214732]*	1,07	-1,86	-2,01
dao.3	*D-amino-acid oxidase 3 [NM_205694]*	-1,36	-11,97	-28,17
dbt	*dihydrolipoamide branched chain transacylase E2 [NM_001013515]*	-1,05	-2,23	-1,92
dcbld1	*discoidin, CUB and LCCL domain containing 1 [NM_001039977]*	3,15	-3,35	-4,33

ddost	*dolichyl-diphosphooligosaccharide-protein glycosyltransferase [NM_213093]*	-1,17	-2,03	-2,19
ddt	*D-dopachrome tautomerase [NM_001002147]*	1,19	-1,85	-1,82
dedd1	*death effector domain-containing 1 [NM_131602]*	1,35	-1,52	-1,03
deptor	*DEP domain containing MTOR-interacting protein [NM_001077320]*	1,31	-1,64	-1,42
dgcr2	*DiGeorge syndrome critical region gene 2 [NM_001002656]*	1,24	-2,75	-1,82
dhdhl	*dihydrodiol dehydrogenase (dimeric), like [NM_214735]*	1,07	-2,81	-2,56
dhrs3b	*dehydrogenase/reductase (SDR family) member 3b[NM_001006070]*	1,01	-1,99	-1,50
dhtkd1	*dehydrogenase E1 and transketolase domain containing 1 [NM_001008619]*	1,14	-2,02	-1,57
dio1	*deiodinase, iodothyronine, type I [NM_001007283]*	1,54	-2,24	-1,85
dio2	*deiodinase, iodothyronine, type II [NM_212789]*	1,48	-7,58	-4,87
dnase1	*deoxyribonuclease I [NM_001002674]*	1,97	1,01	-2,07
dnmt6	*DNA (cytosine-5-)-methyltransferase 6 [NM_001018140]*	1,14	-2,75	-2,20
dpp3	*dipeptidylpeptidase 3 [NM_001002683]*	1,05	-1,93	-1,66
dpys	*dihydropyrimidinase [NM_001123063]*	1,46	-2,00	-1,49
echs1	*enoyl Coenzyme A hydratase, short chain, 1, mitochondrial [NM_001004529]*	1,10	-1,83	-1,95
ecrg4a	*esophageal cancer related gene 4a [NM_001017697]*	1,24	-1,88	-3,73
eef2k	*elongation factor-2 kinase [NM_001002740]*	1,05	-1,91	-1,98
eif3jb	*eukaryotic translation initiation factor 3, subunit Jb [NM_213502]*	-1,28	-1,67	-2,13
eif4bb	*eukaryotic translation initiation factor 4Bb [NM_214718]*	1,28	-1,46	-1,65
elovl2	*elongation of very long chain fatty acids (FEN1/Elo2, SUR4/Elo3, yeast)-like 2 [NM_001040362]*	1,14	-2,88	-3,66
eno1	*enolase 1, (alpha) [NM_212722]*	1,83	1,12	-1,11
ephx1l	*epoxide hydrolase 1, like [XM_001920896]*	1,44	-2,45	-1,95
esf1	*ESF1, nucleolar pre-rRNA processing protein, homolog (S. cerevisiae) [BC080208]*	-1,44	-1,91	-2,12
esr2a	*estrogen receptor 2a [NM_180966]*	1,47	-3,16	-1,68
esr2b	*estrogen receptor 2b [NM_174862]*	-1,03	-2,07	-2,06
eya1	*eyes absent homolog 1 [NM_131193]*	1,38	-3,08	-1,29
f7i	*coagulation factor VIIi [NM_173228]*	1,75	-1,89	-1,88
fabp7a	*fatty acid binding protein 7, brain, a [NM_131605]*	-1,06	-4,46	-4,37
fads2	*fatty acid desaturase 2 [NM_131645]*	1,39	-1,93	-1,63

fahd1	*fumarylacetoacetate hydrolase domain containing 1 [NM_001020728]*	1,15	-2,35	-2,00
fam102ab	*family with sequence similarity 102, member Ab [NM_001076613]*	-1,38	-2,27	-1,56
fam49ba	*family with sequence similarity 49, member Ba [NM_200070]*	-1,24	-2,35	-2,31
fb06f03	*high-mobility group nucleosome binding domain 1-like [NM_001161336]*	1,09	-2,13	-1,64
fep15	*fep15 selenoprotein [NM_001195784]*	1,70	-1,15	-2,52
fgf19	*fibroblast growth factor 19 [NM_001012246]*	-1,40	-25,84	-9,45
fgfr4	*fibroblast growth factor receptor 4 [NM_131430]*	1,11	-2,99	-3,04
fgfrl1a	*fibroblast growth factor receptor-like 1a [NM_200376]*	-1,60	-4,03	-3,97
fkbp1b	*FK506 binding protein 1b [NM_200812]*	1,35	-2,11	-3,16
fkbp2	*FK506 binding protein 2 [NM_001004677]*	-1,39	-1,98	-2,40
fstl1a	*follistatin-like 1a [NM_001017860]*	-1,54	-7,80	-5,86
ftcd	*formiminotransferase cyclodeaminase [NM_201077]*	1,30	-1,57	-1,67
ftr14	*finTRIM family, member 14 [NM_001045270]*	1,19	-1,96	-1,58
ftr53	*finTRIM family, member 53 [NR_037706]*	-3,10	-2,81	-7,10
gamt	*guanidinoacetate N-methyltransferase [NM_001105595]*	1,23	-1,59	-1,74
gart	*phosphoribosylglycinamide formyltransferase [NM_131617]*	-1,28	-2,31	-1,86
gatm	*glycine amidinotransferase (L-arginine:glycine amidinotransferase) [NM_199531]*	1,20	-16,73	-10,93
gb:bm036580	*gb:bm036580 [EH434983]*	1,21	-2,30	-1,82
gbp	*glycogen synthase kinase binding protein [NM_131442]*	1,64	-1,85	-1,44
gbp1	*guanylate binding protein 1[NM_001002343]*	1,78	-1,37	-1,68
gcdh	*glutaryl-Coenzyme A dehydrogenase [NM_200886]*	1,41	-1,78	-1,50
gch2	*GTP cyclohydrolase 2 [NM_131667]*	1,19	-2,07	-2,10
gchfr	*GTP cyclohydrolase I feedback regulator [NM_200762]*	1,40	-4,09	-3,02
ghra	*growth hormone receptor a [NM_001083578]*	1,41	-2,40	-2,50
gmppaa	*GDP-mannose pyrophosphorylase Aa [NM_001002196]*	1,05	-1,97	-1,62
gne	*glucosamine (UDP-N-acetyl)-2-epimerase/N-acetylmannosamine kinase [NM_200883]*	-1,18	-1,92	-2,60
got2a	*glutamic-oxaloacetic transaminase 2a, mitochondrial (aspartate aminotransferase 2) [NM_213379]*	1,18	-1,98	-1,58
gpcpd1	*glycerophosphocholine phosphodiesterase GDE1 homolog (S. cerevisiae) [NM_001110523]*	-1,41	-3,42	-3,02

gpx4b	*glutathione peroxidase 4b [NM_001030070]*	1,01	-2,02	-1,69
grb2	*growth factor receptor-bound protein 2 [NM_213035]*	-1,19	-1,98	-2,13
gspt1	*G1 to S phase transition 1 [NM_001003992]*	-1,12	-2,18	-2,23
gstal	*glutathione S-transferase, alpha-like [NM_213394]*	1,71	-2,03	-2,76
gstm	*glutathione S-transferase M (gstm), mRNA [NM_212676]*	-1,06	-1,89	-2,40
gstm3	*glutathione S-transferase M3 (brain) [NM_001162851]*	1,18	-1,41	-2,18
h3f3c	*H3 histone, family 3C [NM_200003]*	1,53	-1,43	-1,60
hacl1	*2-hydroxyacyl-CoA lyase 1 [NM_213085]*	1,15	-2,27	-1,78
hadh	*hydroxyacyl-Coenzyme A dehydrogenase [NM_001003515]*	1,09	-2,36	-1,93
her9	*hairy-related 9 [NM_131873]*	1,23	-1,18	-1,73
hgd	*homogentisate 1,2-dioxygenase [NM_152966]*	1,29	-1,56	-1,65
hint1	*histidine triad nucleotide binding protein 1 [NM_001005593]*	-1,14	-2,36	-2,27
hmga1b	*high mobility group AT-hook 1b [NM_001077276]*	1,12	-2,83	-1,32
hmga2	*high mobility group AT-hook 2 [NM_212680]*	1,62	-2,00	1,64
hmox1	*heme oxygenase (decycling) 1 [NM_001127516]*	1,26	-1,38	-3,59
hoga1	*4-hydroxy-2-oxoglutarate aldolase 1 [NM_212870]*	1,13	-2,91	-2,82
homer1	*homer homolog 1 (Drosophila) [NM_001002496]*	-1,75	-3,97	-2,20
hp1bp3	*heterochromatin protein 1, binding protein 3 [NM_001199725]*	1,18	-2,53	-2,79
hsd17b4	*hydroxysteroid (17-beta) dehydrogenase 4 [NM_200136]*	1,06	-2,35	-2,04
idh1	*isocitrate dehydrogenase 1 (NADP+), soluble [NM_201499]*	1,20	-1,67	-1,52
ido1	*indoleamine 2,3-dioxygenase 1 [NM_001083854]*	1,22	-1,70	-1,29
ifrd1	*interferon-related developmental regulator 1 [NM_001076555]*	-1,09	-2,12	-2,30
igf2a	*insulin-like growth factor 2a [NM_131433]*	1,55	-2,78	-2,01
igf2b	*insulin-like growth factor 2b [NM_001001815]*	1,38	-3,22	-2,20
igfbp2a	*insulin-like growth factor binding protein 2a [NM_131458]*	1,91	-1,65	-1,03
im:7159406	*im:7159406 [CK694681]*	1,20	-1,85	-1,18
impdh1b	*inosine 5'-phosphate dehydrogenase 1b [NM_001014369]*	1,01	-1,53	-2,22
inhbab	*inhibin, beta Ab [NM_001018156]*	-1,29	-15,86	-56,93
irf11	*interferon regulatory factor 11 [NM_205747]*	-1,00	-3,26	-2,16
irgf1	*immunity-related GTPase family, f1 [NM_001114698]*	1,15	-1,87	-1,32

jag1b	*jagged 1b [NM_131863]*	-1,60	-2,83	-2,53
klf11a	*Kruppel-like factor 11a [NM_001044941]*	1,30	-1,76	-1,88
klhdc4	*kelch domain containing 4 [NM_001126430]*	-1,30	-1,94	-2,00
lgals3bpa	*lectin, galactoside-binding, soluble, 3 binding protein a [NM_001040041]*	1,88	-2,63	-1,42
lhfp	*lipoma HMGIC fusion partner [NM_001008653]*	-1,15	-2,52	-1,59
lin7c	*lin-7 homolog C (C. elegans) [NM_001004672]*	-1,32	-2,43	-1,98
lmf2a	*lipase maturation factor 2a [NM_001128764]*	-1,33	-2,56	-1,68
LOC100000126	*hypothetical LOC100000126 [NM_001130075]*	-1,60	-1,57	-2,21
LOC100003898	*hypothetical LOC100003898 [NM_001100085]*	-1,39	-2,02	-1,79
LOC100005455	*hypothetical LOC100005455 [NM_001161495]*	-1,26	-2,35	-2,13
LOC100006238	*novel protein similar to aldehyde dehydrogenase 9 family, member A1a [NM_001126480]*	1,48	-1,92	-2,50
LOC100148013	*hypothetical LOC100148013 [BC152504]*	-1,25	-2,26	-2,86
LOC100148281	*hypothetical LOC100148281 [NM_001167958]*	-1,34	-1,89	-2,13
LOC100149074	*hypothetical LOC100149074 [BC133147]*	-1,27	-2,13	-1,32
LOC100149918	*hypothetical LOC100149918 [XM_001923533]*	-1,30	-5,08	-4,48
LOC100151204	*Cyp2x12 protein-like [XM_001921268]*	1,29	-4,67	-2,97
LOC556254	*hypothetical LOC556254 [BC142821]*	-1,33	-5,02	-3,59
LOC557314	*similar to chromosome 9 open reading frame 89[BC045390]*	1,09	-3,10	-1,73
LOC559575	*hypothetical LOC559575 [XM_682931]*	1,37	-1,98	-1,10
LOC560297	*hypothetical protein LOC560297 [NM_001126396]*	-1,48	-2,90	-5,40
LOC563048	*similar to hyaluronic acid binding protein 2 [BC122335]*	1,22	-1,96	-2,04
LOC564833	*hypothetical LOC564833 [XM_700557]*	1,36	-1,67	-1,50
LOC567537	*chemokine CXCL-C5c [NM_001115055]*	1,39	-1,73	-1,68
LOC568087	*hypothetical LOC568087 [NM_001145599]*	1,20	-1,99	-1,34
LOC568900	*hypothetical LOC568900 [NR_015620]*	1,60	-1,26	-3,09
LOC569184	*hypothetical protein [CK360688]*	1,05	-2,24	-1,55
LOC792433	*osteocalcin [NM_001083857]*	1,37	-1,84	-1,45
LOC792757	*hypothetical protein LOC792757 [NM_001145701]*	1,21	-1,63	-1,91
LOC794040	*similar to collectin sub-family member 10 [BC095852]*	1,29	-1,59	-1,43

LOC796384	*hypothetical protein LOC796384 [NM_001126459]*	-1,55	-3,05	-2,34
LOC796447	*hypothetical protein LOC796447 [NM_001100056]*	2,02	-1,58	-2,08
LOC799067	*hypothetical LOC799067 [BC127401]*	1,79	-1,15	1,64
lrata	*lecithin retinol acyltransferase a*			
	(phosphatidylcholine--retinol O-acyltransferase a) [NM_001204131]	-1,05	-2,98	-2,78
lrrc17	*leucine rich repeat containing 17 [NM_205539]*	1,35	-5,11	-5,32
lycat	*lysocardiolipin acyltransferase [NM_213270]*	1,01	-2,16	-1,46
lyrm1	*LYR motif containing 1 [NM_001002506]*	-1,28	-1,98	-2,18
lyrm5a	*LYR motif containing 5a [NM_001159823]*	1,23	-1,75	-1,38
lztfl1	*leucine zipper transcription factor-like 1 [NM_199587]*	1,12	-1,84	-1,52
mab21l1	*mab-21-like 1 [NM_152974]*	1,44	-1,66	2,60
magt1	*magnesium transporter 1 [NM_199700]*	1,26	-1,85	-1,80
mak16	*MAK16 homolog (S. cerevisiae) [NM_173239]*	-1,48	-2,38	-3,02
mao	*monoamine oxidase [NM_212827]*	1,57	-1,42	-1,01
map1lc3a	*microtubule-associated protein 1 light chain 3 alpha [NM_214739]*	1,25	-1,60	-1,88
map2k6	*mitogen-activated protein kinase kinase 6 [NM_131724]*	1,15	-1,74	-1,57
map3k12	*mitogen-activated protein kinase kinase kinase 12 [NM_207094]*	-1,84	-3,55	-2,64
map3k4	*mitogen activated protein kinase kinase kinase 4 [AA497200]*	-1,25	-2,55	-1,25
marveld2b	*MARVEL domain containing 2b [NM_001126406]*	-1,56	-1,58	-2,15
mb	*myoglobin [NM_200586]*	1,38	-2,17	-2,87
mccc2	*methylcrotonoyl-Coenzyme A carboxylase 2 (beta) [NM_212927]*	1,20	-1,76	-1,39
mcfd2	*multiple coagulation factor deficiency 2 [NM_001005939]*	-1,39	-1,80	-2,12
mcm3	*MCM3 minichromosome maintenance deficient 3 (S. cerevisiae) [NM_212567]*	-1,40	-3,19	-2,45
mfsd2ab	*major facilitator superfamily domain containing 2ab [NM_001003570]*	1,01	-2,83	-3,06
mgst1	*microsomal glutathione S-transferase 1 [NM_001005957]*	1,20	-2,20	-1,60
miox	*myo-inositol oxygenase [NM_001030266]*	1,15	-6,45	-2,31
mki67ip	*mki67 (FHA domain) interacting nucleolar phosphoprotein (human) [NM_173288]*	-1,69	-1,49	-2,34
mogat3a	*monoacylglycerol O-acyltransferase 3a [NM_001122623]*	1,04	-2,18	-2,10
mpi	*heterochromatin protein 1, binding protein 3 [AI544971]*	1,18	-2,53	-2,79
mpx	*myeloid-specific peroxidase [NM_212779]*	1,27	-2,54	-1,81

mrpl36	*mitochondrial ribosomal protein L36 [BC129237]*	1,01	-2,72	-3,61
ms4a17a.11	*membrane-spanning 4-domains, subfamily A, member 17A.11 [NM_001017670]*	1,55	-1,13	-1,49
msxa	*muscle segment homeobox A [NM_001030269]*	-1,58	-2,85	-1,98
mthfd1	*methylenetetrahydrofolate dehydrogenase (NADP+ dependent) [NM_199529]*	1,26	-2,09	-1,53
mthfsd	*methenyltetrahydrofolate synthetase domain containing [NM_001045099]*	-1,29	-3,72	-4,01
mtor	*mechanistic target of rapamycin (serine/threonine kinase) [NM_001077211]*	1,19	-1,95	-1,73
mtr	*5-methyltetrahydrofolate-homocysteine methyltransferase[NM_198072]*	1,01	-1,91	-2,03
mul1a	*mitochondrial E3 ubiquitin ligase 1a [NM_001018124]*	1,07	-1,92	-1,69
naa20	*N(alpha)-acetyltransferase 20, NatB catalytic subunit [NM_001014329]*	1,24	-1,45	-1,82
ncor1	*nuclear receptor co-repressor 1 [NM_200276]*	1,29	-2,21	-1,54
ndufa4	*NADH dehydrogenase (ubiquinone) 1 alpha subcomplex, 4 [NM_213025]*	-1,18	-9,53	-4,34
nfia	*nuclear factor I/A [NM_001079962]*	1,24	-1,68	-1,75
nit2	*nitrilase family, member 2 [NM_205611]*	1,13	-2,05	-1,50
nots	*nothepsin [NM_131804]*	-1,11	-1,98	-3,82
npm1	*nucleophosmin 1 [NM_199428]*	-1,39	-2,18	-2,23
nr1d2b	*nuclear receptor subfamily 1, group D, member 2b [NM_131065]*	1,28	-1,88	-1,39
nr5a2	*nuclear receptor subfamily 5, group A, member 2 [NM_131463]*	-1,14	-1,84	-2,24
nucb2b	*nucleobindin 2b [NM_201479]*	-1,12	-1,75	-2,67
or111-11	*odorant receptor, family D, subfamily 111, member 11 [NM_131744]*	-1,59	-4,47	-1,22
otub1	*OTU domain, ubiquitin aldehyde binding 1 [NM_001002500]*	1,16	-2,01	-1,94
oxct1a	*3-oxoacid CoA transferase 1a [NM_001007291]*	1,30	-1,60	-1,46
oxct1b	*3-oxoacid CoA transferase 1b [NM_001077150]*	1,74	-1,90	-1,57
oxsr1b	*oxidative-stress responsive 1b [NM_201175]*	-2,28	-1,81	-3,26
paqr5b	*progestin and adipoQ receptor family member Vb [NM_001003573]*	1,18	-3,13	-1,88
park7	*parkinson disease (autosomal recessive, early onset) 7 [NM_001005938]*	-1,06	-2,07	-1,80
parp3	*poly (ADP-ribose) polymerase family, member 3 [NM_200501]*	1,41	-1,89	-1,49
pbp	*phosphatidylethanolamine binding protein [NM_213323]*	1,13	-3,32	-4,31
pcmt	*l-isoaspartyl protein carboxyl methyltransferase [NM_131465]*	-1,00	-2,29	-2,02
pdcd11	*programmed cell death 11 [NM_001089368]*	-1,37	-2,16	-2,34
pdcd4a	*programmed cell death 4a [NM_212988]*	1,43	-1,84	-1,36

pde6g	*phosphodiesterase 6G, cGMP-specific, rod, gamma [NM_212799]*	1,22	-1,83	-1,63
per1b	*period homolog 1b (Drosophila) [NM_212439]*	-1,29	-2,44	-2,74
pex26	*peroxisomal biogenesis factor 26 [NM_199920]*	-1,19	-1,89	-2,19
pfn2l	*profilin 2 like [NM_201466]*	-1,66	-2,34	-2,57
pgk1	*phosphoglycerate kinase 1 [NM_213387]*	1,47	-1,36	-1,32
pglyrp6	*peptidoglycan recognition protein 6 [NM_001045222]*	2,43	-1,52	-3,23
phyhd1	*phytanoyl-CoA dioxygenase domain containing 1 [NM_001007445]*	1,04	-2,68	-2,83
pid1	*phosphotyrosine interaction domain containing 1 [NM_001013504]*	1,26	-2,69	-2,94
pklr	*pyruvate kinase, liver and RBC [NM_201289]*	1,69	-2,00	-3,33
pla1a	*phospholipase A1 member A [NM_207056]*	-1,07	-2,65	-1,69
pnp4b	*purine nucleoside phosphorylase 4b [NM_205643]*	1,48	-1,85	-2,39
polr3gla	*polymerase (RNA) III (DNA directed) polypeptide G like a [NM_001004639]*	-1,17	-2,19	-3,09
pparab	*peroxisome proliferator activated receptor alpha b [NM_001102567]*	1,12	-1,88	-1,69
ppib	*peptidylprolyl isomerase B (cyclophilin B) [NM_213019]*	1,07	-3,26	-3,46
ppp1r14ba	*protein phosphatase 1, regulatory (inhibitor) subunit 14Ba [NM_001045853]*	-1,05	-3,32	-1,82
prdx2	*peroxiredoxin 2 [NM_001002468]*	1,43	-1,26	-1,57
prdx4	*peroxiredoxin 4 [NM_001089425]*	-1,23	-2,56	-2,98
prhoxnb	*parahox cluster neighbor [EH514775]*	1,45	-1,89	-2,11
prickle1b	*prickle homolog 1 (Drosophila) b [NM_001030098]*	-1,67	-4,15	-1,49
prmt3	*protein arginine methyltransferase 3 [NM_001017655]*	-1,37	-1,81	-2,11
psme1	*proteasome activator subunit 1 [NM_131375]*	1,06	-1,90	-1,64
pter	*phosphotriesterase related [NM_001045381]*	1,46	-2,05	-1,74
ptmaa	*prothymosin, alpha a [NM_194376]*	1,15	-2,38	-3,19
ptpn11b	*protein tyrosine phosphatase, non-receptor type 11, b [NM_200454]*	-1,11	-3,52	-2,70
pwp1	*PWP1 homolog (S. cerevisiae) [NM_200292]*	-1,41	-2,13	-1,96
qdprb1	*quinoid dihydropteridine reductase b1 [NM_001020698]*	-1,26	-2,20	-1,87
rab30	*RAB30, member RAS oncogene family [NM_001002750]*	1,00	-2,02	-1,53
rab34	*RAB34, member RAS oncogene family [NM_001006094]*	-1,06	-2,42	-1,98
rbbp9	*retinoblastoma binding protein 9 [NM_001042748]*	-1,25	-1,91	-2,06
rbks	*ribokinase [NM_001002117]*	1,02	-2,33	-2,18

rbp2a	*retinol binding protein 2a, cellular [BC150457]*	1,10	-1,59	-1,84
rbp2b	*retinol binding protein 2b, cellular [CN173643]*	-1,16	-2,05	-2,24
retsat	*retinol saturase (all-trans-retinol 13,14-reductase)[NM_001015061]*	1,04	-2,61	-2,59
rgn	*regucalcin [NM_205746]*	1,43	-2,04	-2,05
rgrb	*retinal G protein coupled receptor b [NM_001024436]*	1,18	-2,18	-2,03
rh50	*Rh50-like protein [NM_131547]*	-1,07	-9,35	-7,65
rhd	*Rh blood group, D antigen [BC115070]*	1,54	-2,18	-1,48
rheb	*ras homolog enriched in brain [NM_001076748]*	-1,12	-2,02	-2,00
rnd1l	*Rho family GTPase 1 like [NM_001044396]*	-1,08	-2,42	-2,09
rorab	*RAR-related orphan receptor A, paralog b [NM_201067]*	1,21	-1,68	-1,18
rplp2l	*ribosomal protein, large P2, like [CT671218]*	-2,49	-3,18	-3,17
rpn1	*ribophorin I [NM_197934]*	1,12	-1,33	-1,80
rpn2	*ribophorin II [NM_212748]*	-1,24	-1,93	-2,46
rpz3	*rapunzel 3 [NM_001111235]*	-1,01	-2,16	-2,46
rrnad1	*ribosomal RNA adenine dimethylase domain containing 1 [NM_001089434]*	-1,02	-1,63	-2,05
sb:cb122	*sb:cb122 [BM259619]*	-1,13	-2,21	-2,18
sb:cb311	*sb:cb311 [CK697995]*	1,26	-2,04	-1,81
sccpdhb	*saccharopine dehydrogenase b [NM_001005574]*	1,22	-1,74	-1,47
scd	*stearoyl-CoA desaturase (delta-9-desaturase) [NM_198815]*	-1,21	-7,95	-15,57
scn4bb	*sodium channel, voltage-gated, type IV, beta b [NM_001077573]*	1,21	-2,03	-1,10
selj	*selenoprotein J [NM_001193469]*	1,36	-1,76	-1,91
sepp1a	*selenoprotein P, plasma, 1a [NM_178297]*	1,15	-1,75	-1,63
sepw1	*selenoprotein W, 1 [NM_178287]*	1,26	-1,70	-1,60
sepw2a	*selenoprotein W, 2a [NM_194417]*	1,24	-1,92	-1,52
serhl	*serine hydrolase-like [NM_199615]*	-1,29	-1,91	-2,04
serpina1	*serine (or cysteine) proteinase inhibitor, clade A (alpha-1 antiproteinase, antitrypsin), member 1 [NM_001077758]*	1,39	-1,66	-1,39
serpinf1	*serine (or cysteine) peptidase inhibitor, clade F, member 1 [NM_001004539]*	1,88	-1,89	-1,94
sfpq	*splicing factor proline/glutamine rich (polypyrimidine tract binding protein associated) [NM_213278]*	1,25	-1,59	-1,70

si:busm1-40g1.2	*si:busm1-40g1.2 [BI888020]*	-1,18	-2,27	-2,10
si:ch211-117n7.7	*si:ch211-117n7.7 [NM_001045343]*	-1,11	-2,65	-2,17
si:ch211-12e1.4	*si:ch211-12e1.4 [NM_001111197]*	1,27	-1,43	-1,73
si:ch211-133n4.4	*si:ch211-133n4.4 [NM_001077376]*	1,16	-1,76	-1,81
si:ch211-160k22.1	*si:ch211-160k22.1 [NM_001127256]*	-1,31	-1,89	-2,26
si:ch211-175f11.3	*si:ch211-175f11.3 [BC090703]*	-1,88	-2,67	-2,55
si:ch211-185o22.2	*si:ch211-185o22.2 [XM_001338801]*	1,28	-2,46	-1,80
si:ch211-193k19.2	*si:ch211-193k19.2 [NM_001083088]*	1,01	-1,85	-2,54
si:ch211-198n5.11	*si:ch211-198n5.11 [NM_001113652]*	1,26	-1,88	-1,14
si:ch211-234f14.1	*si:ch211-234f14.1 [NM_001144053]*	1,16	-1,37	1,56
si:ch211-234p6.12	*si:ch211-234p6.12[NM_001114915]*	1,24	-2,30	-2,53
si:ch211-240l19.5	*si:ch211-240l19.5 [NM_001030152]*	1,64	1,17	-1,92
si:ch211-240l19.6	*si:ch211-240l19.6 [NM_001082928]*	1,77	-1,46	-3,48
si:ch211-240l19.7	*si:ch211-240l19.7 [NM_001082929]*	1,48	-1,05	-2,63
si:ch211-240l19.8	*si:ch211-240l19.8 [NM_001082930]*	1,75	1,24	-1,67
si:ch211-284e20.8	*si:ch211-284e20.8 [NM_001099265]*	1,24	-2,27	-1,57
si:ch211-42i9.8	*si:ch211-42i9.8 [NM_001145618]*	-1,32	-2,03	-1,77
si:ch211-5k11.6	*si:ch211-5k11.6 [NM_001013461]*	1,66	-1,93	-2,53
si:ch211-89p1.1	*si:ch211-89p1.1 [NM_001082982]*	1,17	-2,52	-1,87
si:ch73-131e21.5	*si:ch73-131e21.5 [BC122309]*	1,36	-3,26	-4,64
si:ch73-15j19.1	*si:ch73-15j19.1 [NM_001005604]*	-3,37	-4,91	-1,28
si:ch73-252g14.4	*si:ch73-252g14.4 [NM_001100029]*	2,03	-1,88	-3,24
si:ch73-46j18.5	*si:ch73-46j18.5 [NR_036690]*	1,12	-4,50	-4,71
si:dkey-149j18.2	*si:dkey-149j18.2 [NM_001044953]*	-1,14	-1,72	-2,22
si:dkey-14d8.7	*si:dkey-14d8.7 [NM_001044836]*	1,36	-2,50	-6,85
si:dkey-14k9.3	*si:dkey-14k9.3 [NM_001039063]*	-1,19	-2,22	-1,62
si:dkey-151c10.1	*si:dkey-151c10.1 [NM_001100032]*	-1,02	-2,14	-1,99
si:dkey-236e20.2	*si:dkey-236e20.2 [NM_001082876]*	1,04	-2,26	-2,48
si:dkey-239i20.2	*si:dkey-239i20.2 [NM_001044829]*	1,18	-1,73	-1,62
si:dkey-37m8.9	*si:dkey-37m8.9 [XM_686251]*	1,93	-2,56	-2,06

si:dkeyp-35f11.3	*si:dkeyp-35f11.3 [NM_001122973]*	2,33	-1,85	-1,89
si:rp71-15k1.1	*si:rp71-15k1.1 [NM_001079945]*	1,43	-1,72	-1,40
si:xx-by187g17.1	*si:xx-by187g17.1 [NM_001033093]*	1,29	-1,51	-1,59
sin3aa	*SIN3 homolog A, transcription regulator (yeast) a [NM_001098180]*	-1,50	-2,29	-2,00
skia	*nuclear oncoprotein skia [NM_130935]*	1,29	-1,99	-1,97
slc16a12b	*solute carrier family 16 (monocarboxylic acid transporters), member 12b[NM_001145814]*	1,16	-3,25	-5,19
slc16a4	*solute carrier family 16 (monocarboxylic acid transporters), member 4 [NM_001080599]*	1,07	-3,45	-3,73
slc25a27	*solute carrier family 25, member 27 [NM_200341]*	-1,22	-2,59	-2,03
slc25a32a	*solute carrier family 25, member 32a [NM_200256]*	1,43	-2,54	-2,88
slc2a5	*solute carrier family 2 (facilitated glucose/fructose transporter), member 5 [NR_023322]*	-1,13	-3,29	-2,14
slc31a1	*solute carrier family 31 (copper transporters), member 1 [NM_205717]*	-1,31	-2,60	-2,09
slc43a1b	*solute carrier family 43, member 1b [NM_001083000]*	1,63	-3,63	-3,46
slc47a1	*solute carrier family 47, member 1 [NM_001014310]*	1,22	-2,42	-1,59
smad7	*MAD, mothers against decapentaplegic homolog 7 (Drosophila) [NM_175082]*	1,77	-1,75	-1,57
sox11a	*SRY-box containing gene 11a [NM_131336]*	1,34	-1,17	-1,81
spam1	*sperm adhesion molecule 1 [NM_001080685]*	2,80	-3,44	-1,00
sparc	*secreted acidic cysteine rich glycoprotein[NM_001001942]*	1,32	-1,74	-1,81
sprn	*shadow of prion protein [NM_198981]*	1,21	-3,23	1,02
srd5a2a	*steroid-5-alpha-reductase, alpha polypeptide 2a [NM_001017703]*	-1,08	-2,69	-2,37
srp19	*signal recognition particle 19 [NM_213343]*	-1,26	-2,17	-3,18
ssr1	*signal sequence receptor, alpha [NM_201327]*	-1,14	-2,05	-2,77
stat5.1	*signal transducer and activator of transcription 5.1 [NM_194387]*	1,15	-1,77	-1,21
stim1b	*stromal interaction molecule 1b [NM_001202541]*	1,43	-3,01	-1,42
sult1st3	*sulfotransferase family 1, cytosolic sulfotransferase 3 [NM_183348]*	-1,50	-2,27	-3,23
sult1st6	*sulfotransferase family 1, cytosolic sulfotransferase 6 [NM_001002599]*	-1,02	-2,50	-1,73
sult3st2	*sulfotransferase family 3, cytosolic sulfotransferase 2 [NM_001079947]*	1,28	-2,57	-2,47
taldo1	*transaldolase 1 [BC068191]*	1,37	-1,83	-3,01

tat	*tyrosine aminotransferase [NM_001077554]*	1,35	-1,90	-1,88
tef	*thyrotroph embryonic factor [NM_131400]*	2,10	-2,64	-2,44
tfa	*transferrin-a [NM_001015057]*	1,43	-2,10	-1,55
tfap2b	*transcription factor AP-2 beta [NM_001024665]*	-1,26	-3,20	-4,48
tgfbi	*transforming growth factor, beta-induced [NM_182862]*	1,04	-2,48	-2,21
thbs3a	*thrombospondin 3a [NM_173225]*	1,15	-1,78	-1,44
thnsl2	*threonine synthase-like 2 (S. cerevisiae) [NM_001037566]*	1,48	-2,49	-2,04
thrb	*thyroid hormone receptor beta [NM_131340]*	1,26	-1,89	-1,42
tlr3	*toll-like receptor 3 [NM_001013269]*	1,13	-2,61	-1,66
tm4sf5	*transmembrane 4 L six family member 5 [NM_001002372]*	1,11	-2,24	-1,84
tmed1a	*transmembrane emp24 protein transport domain containing 1a [NM_001003487]*	-1,46	-2,22	-2,06
tmem206	*transmembrane protein 206 [NM_200457]*	1,12	-1,98	-2,20
tmem56b	*transmembrane protein 56b [NM_001005924]*	-1,27	-3,14	-2,31
tmem64	*transmembrane protein 64 [NM_001128251]*	-1,56	-2,93	-2,51
tmem88b	*transmembrane protein 88 b [NM_001077144]*	1,21	-1,97	-1,84
tnr	*tenascin R (restrictin, janusin) [NM_194383]*	1,14	-2,02	-1,58
tph2	*tryptophan hydroxylase 2 (tryptophan 5-monooxygenase) [NM_214795]*	1,48	-2,04	-2,09
tpk1	*thiamin pyrophosphokinase 1 [NM_001006074]*	1,25	-1,99	-1,64
tpst1	*tyrosylprotein sulfotransferase 1 [NM_131403]*	-1,32	-2,35	-2,16
trim2a	*tripartite motif-containing 2a [NM_001014371]*	1,25	-1,71	-1,21
trim44	*tripartite motif-containing 44 [NM_001161749]*	1,15	-2,04	-1,63
try	*trypsin [NM_131708]*	1,48	1,07	-1,68
tspo	*translocator protein [NM_001006032]*	1,44	-1,73	-1,76
ttc4	*tetratricopeptide repeat domain 4 [NM_201006]*	-1,42	-1,55	-2,20
tuba8l3b	*tubulin alpha 8-like 3b [NM_001168287]*	1,49	-1,29	-1,86
ubtfl	*upstream binding transcription factor, like [NM_201003]*	1,13	-2,17	-1,33
ugt5b6	*UDP glucuronosyltransferase 5 family, polypeptide B6 [NM_001040345]*	1,64	-2,07	-1,39
ugt5c2	*UDP glucuronosyltransferase 5 family, polypeptide C2 [NM_001045386]*	1,42	-2,26	-2,15
ugt5c3	*UDP glucuronosyltransferase 5 family, polypeptide C3 [NM_001128714]*	1,18	-2,14	-1,98
ugt5e1	*UDP glucuronosyltransferase 5 family, polypeptide E1 [NM_001128285]*	1,24	-1,85	-1,17

uox	*urate oxidase [NM_001002332]*	1,63	-2,06	-1,82
upb1	*ureidopropionase, beta [NM_199616]*	1,48	-2,26	-2,07
upp2	*uridine phosphorylase 2 [NM_200144]*	1,22	-1,91	-2,23
wdr89	*WD repeat domain 89 [NM_001167958]*	-1,34	-1,89	-2,13
wu:fa94b01	*wu:fa94b01 [EH454968]*	-1,04	-3,05	-2,31
wu:fb57c04	*wu:fb57c04 [CK028617]*	1,08	-7,96	-3,00
wu:fb73e05	*wu:fb73e05 [AI545005]*	-1,11	-2,27	-1,54
wu:fb81b05	*wu:fb81b05 [CT603629]*	1,06	-3,22	-1,47
wu:fb81c07	*wu:fb81c07 [CF549786]*	-1,48	-1,86	-2,41
wu:fb83c11	*wu:fb83c11 [AI721552]*	-1,06	-2,97	-2,58
wu:fb96e05	*wu:fb96e05 [AI588546]*	1,11	-1,92	-1,70
wu:fc03e06	*wu:fc03e06 [CT650814]*	1,28	-1,83	-1,37
wu:fc13c02	*wu:fc13c02 [AJ245490]*	-1,14	-2,21	-1,63
wu:fc14a10	*wu:fc14a10 [XM_690545]*	1,18	-2,43	-2,72
wu:fc16e03	*wu:fc16e03 [EL651413]*	1,24	-2,06	-1,87
wu:fc25f11	*wu:fc25f11 [AI721960]*	1,68	-1,39	1,65
wu:fc28h03	*wu:fc28h03 [DT075619]*	1,17	-2,42	-1,65
wu:fc41f02	*wu:fc41f02 [AI667541]*	1,64	-1,95	3,34
wu:fc48d03	*wu:fc48d03 [CT711365]*	1,13	-1,77	-1,49
wu:fc51e09	*wu:fc51e09 [CT611256]*	-1,01	-2,86	-2,40
wu:fc55g01	*wu:fc55g01 [CN021867]*	-1,51	-2,52	-2,05
wu:fc65g01	*wu:fc65g01 [CT666954]*	1,01	-2,48	-2,63
wu:fc66h12	*wu:fc66h12 [CT729493]*	-1,05	-2,01	-1,78
wu:fd43b10	*wu:fd43b10 [BC077122]*	1,06	-1,93	-1,66
wu:fd43b11	*wu:fd43b11 [AW019510]*	-1,01	-3,86	-1,50
wu:fd60d11	*wu:fd60d11 [EH999512]*	1,13	-2,07	-1,75
wu:fe11f09	*wu:fe11f09 [CT609156]*	-1,29	-1,82	-2,05
wu:fi71b02	*wu:fi71b02 [BE605427]*	1,15	-2,88	-2,18
wu:fj04f08	*wu:fj04f08 [AW076848]*	-2,22	-1,81	-3,22
wu:fj09e11	*wu:fj09e11 [EH459157]*	1,10	-1,97	-1,65

wu:fk35g07	*wu:fk35g07 [AW567217]*	-1,13	-2,19	-1,84
wu:fk54d01	*wu:fk54d01 [CO926823]*	1,15	-4,56	-2,82
wu:fk86g06	*wu:fk86g06 [EE718204]*	1,25	-3,84	-2,89
wu:fk89d07	*wu:fk89d07 [BE200686]*	1,06	-2,62	-2,01
wu:fk91b07	*wu:fk91b07 [BE200781]*	1,37	-1,85	-2,22
xirp2a	*xin actin-binding repeat containing 2 [XM_683113]*	1,19	-1,89	-1,72
zbtb8os	*zinc finger and BTB domain containing 8 opposite strand [NM_001017687]*	-1,24	-2,78	-2,80
zfp36l1a	*zfp36l1a [CT659468]*	-1,34	-2,27	-2,21
zfyve21	*zinc finger, FYVE domain containing 21 [NM_001160346]*	-1,07	-2,05	-1,89
zgc:101129	*zgc:101129 [NM_001003759]*	1,41	-4,52	-1,50
zgc:101699	*zgc:101699 [NM_001007430]*	1,01	-2,44	-1,72
zgc:101847	*zgc:101847 [NM_001008593]*	1,44	-2,15	-2,03
zgc:103438	*zgc:103438 [NM_001006036]*	1,01	-1,71	-2,04
zgc:103559	*zgc:103559 [NM_001008599]*	1,27	-3,60	-2,81
zgc:103586	*zgc:103586 [NM_001007389]*	1,69	-1,70	-1,01
zgc:103594	*zgc:103594 [NM_001004680]*	1,15	-1,69	-1,92
zgc:103601	*zgc:103601 [NM_001005974]*	1,63	-2,25	-2,04
zgc:103627	*zgc:103627 [NM_001005954]*	1,19	-1,81	-2,03
zgc:103654	*zgc:103654 [NM_001007363]*	1,21	-2,63	-2,62
zgc:110326	*zgc:110326 [NM_001017805]*	1,86	-1,08	2,14
zgc:110339	*zgc:110339 [NM_001017792]*	1,28	-1,58	-1,59
zgc:110388	*zgc:110388 [NM_001017657]*	-1,59	-1,88	-2,26
zgc:110664	*zgc:110664 [NM_001177745]*	-1,21	-2,04	-1,86
zgc:110759	*zgc:110759 [NM_001013316]*	1,41	-2,08	-1,80
zgc:110784	*zgc:110784 [NM_001014366]*	1,42	-2,34	-1,35
zgc:110848	*zgc:110848 [NM_001013329]*	-1,01	-2,03	-1,68
zgc:112000	*zgc:112000 [NM_001017690]*	-1,24	-1,64	-2,04
zgc:112160	*zgc:112160 [NM_001017724]*	1,53	-2,97	-7,87
zgc:112210	*zgc:112210 [NM_001024411]*	1,14	-2,98	-1,78
zgc:112368	*zgc:112368 [NM_001025474]*	2,01	1,06	-1,85

zgc:112374	*zgc:112374 [NM_001024417]*	1,14	-2,02	-1,96
zgc:112384	*zgc:112384 [NM_001020694]*	1,42	-1,45	-1,38
zgc:112407	*zgc:112407 [NM_001020699]*	1,31	-2,35	-1,44
zgc:112519	*zgc:112519 [NM_001017757]*	-1,17	-2,42	-2,04
zgc:114148	*zgc:114148 [NM_001033729]*	1,05	-2,49	-2,52
zgc:123180	*zgc:123180 [NM_001037107]*	-1,33	-2,04	-2,72
zgc:123207	*zgc:123207 [NM_001037408]*	-1,18	-2,10	-1,58
zgc:123280	*zgc:123280 [NM_001037690]*	1,07	-1,94	-1,74
zgc:123333	*zgc:123333 [NM_001037388]*	1,55	-1,27	-1,68
zgc:136360	*zgc:136360 [NM_001040337]*	-1,29	-1,93	-2,27
zgc:136493	*zgc:136493 [NM_001045249]*	1,07	-3,20	-3,30
zgc:136538	*zgc:136538 [NM_001045237]*	1,41	-2,39	-1,36
zgc:136864	*zgc:136864 [NM_001040044]*	1,29	-1,20	-1,93
zgc:136871	*zgc:136871 [NM_001040379]*	-1,21	-3,43	-2,83
zgc:153032	*zgc:153032 [NM_001076638]*	-1,09	-2,13	-1,89
zgc:153148	*zgc:153148 [NM_001076742]*	1,26	-1,58	-2,07
zgc:153177	*zgc:153177 [NM_001172678]*	-1,46	-2,63	-3,03
zgc:153296	*zgc:153296 [NM_001076630]*	1,07	-2,94	-2,58
zgc:153328	*zgc:153328 [EH562186]*	-1,24	-1,82	-2,16
zgc:153507	*zgc:153507 [NM_001077736]*	1,23	-2,09	-1,94
zgc:153610	*zgc:153610 [NM_001076730]*	-1,14	-2,21	-1,53
zgc:153700	*zgc:153700 [BC078329]*	1,06	-1,62	-2,02
zgc:153912	*zgc:153912 [NM_001079971]*	1,09	-2,81	-2,12
zgc:153914	*zgc:153914 [NM_001077720]*	1,09	-1,83	-2,05
zgc:153941	*zgc:153941 [NM_001076607]*	1,35	-1,95	-2,56
zgc:153957	*zgc:153957 [NM_001083851]*	1,16	-1,74	-1,51
zgc:153976	*zgc:153976 [NM_001077733]*	-1,08	-2,10	-1,63
zgc:154006	*zgc:154006 [NM_001080004]*	1,14	-2,12	-2,22
zgc:154169	*zgc:154169 [NM_001077793]*	1,32	-3,93	-3,60
zgc:158258	*zgc:158258 [NM_001080668]*	1,21	-1,89	-1,23

zgc:158309	*zgc:158309 [NM_001079978]*	1,49	-1,45	-1,44
zgc:158350	*zgc:158350 [NM_001080993]*	-1,39	-1,18	-2,40
zgc:158483	*zgc:158483 [BC133104]*	-2,13	-1,80	-3,20
zgc:158628	*zgc:158628 [NM_001080698]*	1,89	1,20	-2,36
zgc:162318	*zgc:162318 [BC134960]*	1,21	-1,82	-1,34
zgc:162356	*zgc:162356 [NM_001045060]*	1,32	-2,47	-2,91
zgc:162396	*zgc:162396 [NM_001044782]*	1,14	-1,90	-1,84
zgc:162816	*zgc:162816 [NM_001089489]*	1,42	-1,80	-1,55
zgc:172049	*zgc:172049 [NM_001113616]*	-1,19	-2,21	-1,48
zgc:172051	*zgc:172051 [NM_001128299]*	1,43	-1,63	-1,07
zgc:172053	*zgc:172053 [NM_001111242]*	1,00	-2,08	-3,08
zgc:172238	*zgc:172238 [NM_001113635]*	1,24	-1,87	-1,42
zgc:172253	*zgc:172253 [NM_001114483]*	-1,35	-6,31	-5,01
zgc:173587	*zgc:173587 [NM_001109836]*	-1,10	-4,31	-7,34
zgc:173617	*zgc:173617 [NM_001109839]*	1,12	-1,82	-1,29
zgc:173994	*zgc:173994 [NM_001110116]*	1,10	-1,72	-2,23
zgc:174260	*zgc:174260 [NM_001114704]*	1,51	-2,37	-2,19
zgc:174637	*zgc:174637 [NM_001105122]*	1,20	-1,74	-1,75
zgc:174646	*zgc:174646 [NM_001122615]*	1,46	-1,52	-1,67
zgc:174650	*zgc:174650 [NM_001114927]*	1,28	-2,22	-1,17
zgc:174895	*zgc:174895 [NM_001105704]*	1,06	-3,81	-4,24
zgc:175107	*zgc:175107 [NM_001113632]*	1,36	-1,58	1,21
zgc:193616	*zgc:193616 [EH506703]*	2,98	-8,03	-11,76
zgc:193682	*zgc:193682 [NM_001135978]*	1,14	-2,04	-2,07
zgc:193725	*zgc:193725 [EH551598]*	1,35	-1,95	-5,13
zgc:194242	*zgc:194242 [NM_001128815]*	1,33	-1,89	-1,75
zgc:195062	*zgc:195062 [NM_001130785]*	-1,22	-2,25	-2,39
zgc:55418	*zgc:55418 [NM_199859]*	1,15	-2,26	-2,48
zgc:56136	*zgc:56136 [NM_200198]*	1,93	-1,53	-1,96
zgc:64012	*zgc:64012 [NM_214761]*	1,30	-2,22	-1,16

zgc:65857	*zgc:65857 [NM_200580]*	1,80	-1,30	-1,25
zgc:65960	*zgc:65960 [NM_200549]*	1,26	-2,24	-1,96
zgc:66313	*zgc:66313 [NM_200428]*	1,24	-1,89	-2,28
zgc:66382	*zgc:66382 [NM_199605]*	1,29	-1,22	-1,97
zgc:73155	*zgc:73155 [NM_200829]*	1,04	-9,61	-5,41
zgc:73210	*zgc:73210 [NM_200749]*	-1,12	-2,39	-2,26
zgc:73311	*zgc:73311 [NM_001193525]*	1,41	-2,28	-2,09
zgc:77041	*zgc:77041 [NM_207100]*	-1,47	-1,68	-2,33
zgc:77058	*zgc:77058 [NM_212900]*	-1,18	-2,29	-1,93
zgc:77158	*zgc:77158 [NM_205672]*	-1,03	-2,50	-3,15
zgc:77513	*zgc:77513 [NM_212677]*	1,16	-1,89	-1,54
zgc:77556	*zgc:77556 [NM_212734]*	-1,03	-3,48	-2,16
zgc:77734	*zgc:77734 [NM_199608]*	1,14	-2,10	-2,14
zgc:77778	*zgc:77778 [NM_213203]*	2,23	-2,00	-2,93
zgc:77938	*zgc:77938 [NM_205642]*	1,06	-5,10	-3,04
zgc:85716	*zgc:85716 [NM_214815]*	1,21	-1,72	-1,33
zgc:85777	*zgc:85777 [NM_212935]*	1,43	-1,49	-1,59
zgc:85789	*zgc:85789 [NM_212616]*	1,46	-1,46	-1,47
zgc:85829	*zgc:85829 [NM_213276]*	-1,33	-2,37	-1,86
zgc:85882	*zgc:85882 [NM_212942]*	-1,15	-2,18	-1,88
zgc:91852	*zgc:91852 [NM_001002197]*	1,26	-1,84	-1,47
zgc:91890	*zgc:91890 [NM_001002182]*	1,14	1,47	-1,45
zgc:91944	*zgc:91944 [NM_001002668]*	1,12	-2,54	1,07
zgc:92040	*zgc:92040 [NM_001002391]*	1,36	-5,01	-3,90
zgc:92041	*zgc:92041 [NM_001003737]*	2,19	-1,00	-2,83
zgc:92249	*zgc:92249 [NM_001002623]*	-1,20	-2,30	-2,59
zgc:92357	*zgc:92357 [NM_001002215]*	1,08	-1,94	-1,66
zgc:92429	*zgc:92429 [NM_001003457]*	1,19	-2,69	-3,29
zgc:92511	*zgc:92511 [NM_001003450]*	1,76	-1,19	-1,83
zgc:92590	*zgc:92590 [NM_001007054]*	1,81	-1,01	-2,27

zgc:92739	*zgc:92739 [BC107641]*	1,26	-2,86	-2,01
zgc:92745	*zgc:92745 [NM_001003426]*	2,66	-1,04	-4,73
zgc:92749	*zgc:92749 [NM_001002570]*	1,82	-2,90	-3,63
zgc:92763	*zgc:92763 [NM_001002560]*	1,20	-4,00	-3,34
zgc:92869	*zgc:92869 [NM_001030271]*	1,15	-1,79	-1,65
zgc:92902	*zgc:92902 [NM_001002462]*	-1,07	-2,07	-1,31
znf395b	*zinc finger protein 395b [NM_001045115]*	-1,25	-2,40	-2,17
znfl1	*zinc finger-like gene 1 [NM_001113633]*	1,26	-1,48	-1,99

Common to packing and transport

**Gene symbol**	**Gene name**	**Fold change***		
		**0 h**	**48 h**	**72 h**
acot7	*acyl-CoA thioesterase 7 [NM_001004617]*	-2,38	-3,00	-3,09
bhlhe41	*basic helix-loop-helix family, member e41 [NM_001039107]*	-1,97	-2,88	-2,62
dap	*death associated protein [NM_131572]*	-1,73	-3,55	-3,28
lgi3	*leucine-rich repeat LGI family, member 3 [NM_001039679]*	-7,74	-5,87	-15,53
lmf2b	*lipase maturation factor 2b [NM_001142563]*	-1,78	-2,10	-2,15
mat2a	*methionine adenosyltransferase II, alpha [NM_212637]*	-2,09	-2,16	-2,49
mid1ip1b	*MID1 interacting protein 1b [NM_200832]*	-2,75	-3,36	-4,04
nola1	*nucleolar protein family A, member 1 (H/ACA small nucleolar RNPs) [NM_200975]*	-1,70	-1,85	-2,06
otud4	*zgc:165536 [BC142849]*	-1,30	-3,13	-2,83
p4ha1b	*procollagen-proline, 2-oxoglutarate 4-dioxygenase (proline 4-hydroxylase), alpha polypeptide I [BC153498]*	-1,65	-2,27	-2,10
pim1	*pim-1 oncogene [NM_131539]*	-3,16	-10,31	-9,63
pisd	*phosphatidylserine decarboxylase [NM_001079702]*	-3,10	-3,95	-4,44
rcn3	*reticulocalbin 3, EF-hand calcium binding domain [NM_001002158]*	-3,57	-3,31	-2,62
si:ch211-15i6.4	*si:ch211-15i6.4 [NM_001044926]*	-1,63	-4,23	-1,87
si:ch211-234p6.13	*si:ch211-234p6.13 [NM_001025297]*	-2,51	-1,05	-1,31

si:ch211-244p18.3	*si:ch211-244p18.3 [NM_001045119]*	-1,00	-1,66	-2,00
si:dkeyp-44b8.8	*si:dkeyp-44b8.8 [NM_001159933]*	-2,26	-1,71	-2,85
slc20a1b	*solute carrier family 20, member 1b [NM_212588]*	-2,18	-1,80	-2,44
slc40a1	*solute carrier family 40 (iron-regulated transporter), member 1 [NM_131629]*	-2,64	-3,88	-3,00
spcs2	*signal peptidase complex subunit 2 homolog (S. cerevisiae) [NM_001013469]*	-1,35	-1,64	-2,09
wu:fc72f01	*wu:fc72f01 [EH487450]*	-1,79	-3,31	-3,45
zgc:101071	*zgc:101071 [NM_001003569]*	-2,54	-4,60	-3,29
zgc:101609	*zgc:101609 [NM_001007455]*	-1,55	-1,45	-2,37
zgc:112026	*zgc:112026 [NM_001017672]*	-6,29	-8,64	-3,75
zgc:123010	*zgc:123010 [NM_001145554]*	-1,78	-2,10	-2,15
zgc:123275	*zgc:123275 [NM_001037412]*	-1,98	-2,34	-1,75
zgc:158228	*zgc:158228 [NM_001080648]*	-3,03	-1,47	-2,42
zgc:158376	*zgc:158376 [NM_001100147]*	-4,37	-2,83	-3,26
zgc:63694	*zgc:63694 [NM_213106]*	-5,80	-7,70	-3,94
zgc:77390	*zgc:77390 [NM_205652]*	-1,62	-2,32	-2,46
zgc:92746	*zgc:92746 [NM_001002572]*	-1,54	-1,66	-2,20

List of genes that are significantly downregulated immediately after packing (0 h), and at 48 and 72 h during transport compared to the expression in liver of fish prior to transport (basal or pre-stressed). * Fold-changes (positive for upregulated and negative for downregulated genes) are calculated as the ratio of the gene expression level at a particular time point to the respective basal levels.
